# Optimization of Microfluidizer-Produced PLGA Nano-Micelles for Enhanced Stability and Antioxidant Efficacy: A Quality by Design Approach

**DOI:** 10.3390/pharmaceutics18010025

**Published:** 2025-12-25

**Authors:** Esma Nur Develi Arslanhan, Fatemeh Bahadori, Zahra Eskandari, Muhammed Zahid Kasapoglu, Erkan Mankan

**Affiliations:** 1 Department of Biotechnology, Institute of Health Sciences, Bezmialem Vakif University, Fatih, 34093 Istanbul, Turkey; esmadeveli595@gmail.com; 2 Department of Analytical Chemistry, Faculty of Pharmacy, Istanbul University-Cerrahpasa, Buyukcekmece Campus, 34500 Istanbul, Turkey; 3 Department of Pharmaceutical Biotechnology, Faculty of Pharmacy, Bezmialem Vakif University, Fatih, 34093 Istanbul, Turkey; zeskandari@mgh.harvard.edu; 4 Department of Chemistry, Biochemistry Division, Yildiz Technical University, 34220 Istanbul, Turkey; 5 Department of Nanotechnology, Institute of Nanotechnology and Biotechnology, Istanbul University-Cerrahpasa, 34500 Istanbul, Turkey; muhammedkasapoglu@entertech.com.tr; 6 Heka Laboratory, Senol Gunes Boulevard, Camlik District, No.34 Heka Plaza, Umraniye, 34764 Istanbul, Turkey; emankan@gmail.com

**Keywords:** quality by design, PLGA, hydrophilic, hydrophobic, microfluidizer, stability, antioxidant

## Abstract

**Introduction:** In this study, we aimed to optimize the microfluidizer-based preparation of poly(lactic-co-glycolic acid) nano-micelles (PLGANM), increasingly used for parenteral delivery of poorly water-soluble drugs but typically exhibiting poor physical stability when produced by conventional methods. **Method:** By systematically tuning microfluidization (MFZ) parameters, we demonstrate an efficient strategy to enhance PLGANM stability and ensure robust, scalable manufacturing, relevant for long-term storage and clinical translation applications. The influence of several key factors designed by Central Composite Design (CCD), including the amount of PLGA and Tween 80, homogenization pressure, and number of passes of MFZ on the size, polydispersity (measured by DLS), and hence stability of the PLGANM, was analyzed for 60 days. 60 PLGANMs produced by the MFZ method (PMFZ) were compared with the PLGANM consisting of equivalent amounts of PLGA and T80 produced using the traditional oil-in-water method (POW). Desired limits were set to minimize standard deviations for Z-average, Zeta Potential, and PDI. **Results:** Coded variables for optimized PMFZ (OPMFZ) were found to be 82.96 mg PLGA, 6.78 mL 5% T80, 11,000 psi pressure, and 1 pass. **Conclusions:** This study demonstrates that microfluidization, when guided by a QbD framework, offers precise control over particle attributes and enables reproducible production of stable PLGANM.

## 1. Introduction

Delivering therapeutic agents specifically to tumor sites requires the development of reliable and efficient drug delivery systems. Polymeric micelles—especially those made of poly (lactic-co-glycolic acid) (PLGA)—have drawn considerable interest as drug delivery vehicles because of their biocompatibility, biodegradability, and capacity to carry both hydrophilic and hydrophobic medications. PLGA can form a variety of supramolecular structures. The structure formed by PLGA mainly depends on the particle formation method. Micellar structures are typically formed by dissolving PLGA in an organic solvent and slowly introducing it into an aqueous medium. This method is often referred to as oil-in-water. Micelles are formed by the escape of the hydrophobic lactide block of PLGA from water and exposing the hydrophilic gluconic acid block to water [1,2].

PLGA can encapsulate hydrophobic drugs into nano-sized micelles, enhancing their solubility and bioavailability while shielding them from degradation. PLGA’s hydrophobic character enables it to form nanoparticles in aqueous environments, and the addition of surfactants like Tween 80 helps stabilize these nanoparticles by forming a hydrophilic shell around a hydrophobic core, effectively encapsulating the drug.

Because of this distinct core–shell structure, PLGA nano-micelles (PLGANM) are a desirable option for targeted drug delivery [3]. However, a major challenge with PLGANM is its stability during storage and transportation. The stability of such nano-micelles can be compromised by physical instability (e.g., aggregation, precipitation), chemical instability (degradation of the polymer or drug), and colloidal instability (e.g., changes in osmotic pressure or surface charge). Addressing these stability issues is crucial for the successful translation of PLGANM to clinical applications. For instance, Alvi et al. [4] highlighted that PLGA-based nanoparticles show promise in cancer therapy but require improved stability for clinical success, and Balibey et al. [5] demonstrated that optimizing a PLGA-DSPE hybrid nano-micelle significantly enhanced curcumin delivery and stability, reinforcing the importance of resolving stability challenges.

Quality by Design (QbD) is a systematic approach to drug development and manufacturing that emphasizes understanding the product and process, and the identification and control of critical quality attributes. In the context of PLGANM, a QbD approach can be employed to optimize the formulation and process to ensure the stability and performance of the final product. QbD involves identifying critical material attributes (CMAs), critical process parameters (CPPs), and critical quality attributes (CQAs) that impact nanoparticle performance. The main goal is to develop detailed process and product understanding so that a robust formulation process can consistently yield the desired quality.

A QbD study typically relies on well-established design-of-experiments (DoE) tools [6]. Conventional one-factor-at-a-time experimentation is inefficient and fails to capture interactions between factors [7]. In contrast, statistical DoE approaches yield more accurate results with fewer runs, allowing mathematical modeling of responses. Numerous experimental designs in the literature can be applied to reduce the number of experiments while obtaining more useful data [8,9]. If the goal is to precisely optimize a process, a response surface design such as Central Composite Design (CCD) is often used. In this study, we employed a CCD for formulation optimization.

By understanding the relationships between these critical factors, a robust formulation and manufacturing process can be developed to consistently produce stable and effective PLGANM. Indeed, previous work has utilized a CCD-based QbD approach for optimizing PLGA nanoparticle formulations [10].

In this study, we explored the potential of Microfluidizer (MFZ) technology to increase the stability of PLGANM, and we implemented a QbD approach to optimize the formulation and manufacturing process.

Microfluidizer technology has emerged as a powerful tool for producing nano-sized drug delivery systems such as PLGANM. Microfluidizers are high-pressure homogenization devices that can effectively reduce particle size and produce homogeneous dispersions. The technology uses high shear forces generated by colliding fluid streams at high velocities, which disrupt larger particles into smaller, uniformly sized ones. Whereas conventional emulsion techniques often yield inconsistent particle sizes and poor stability, microfluidization ensures uniformity and stability through efficient mixing under controlled processing parameters. Additionally, microfluidizer technology enables scalability without compromising product quality, making it suitable for industrial-scale production of stable PLGANM.

In the context of PLGANM formation, microfluidizer processing offers precise control over particle size and distribution, leading to improved nanoparticle stability and drug-loading capacity. This improvement has been demonstrated in multiple studies: Operti et al. [11,12] used continuous microfluidization to produce uniformly sub-micron PLGA particles; Sani et al. [13] showed that adjusting microfluidization parameters (such as pressure and cycle number) alters the size and polydispersity of PEG-PLGA nanoparticles; Operti et al. [12] reviewed PLGA nanomedicine manufacturing techniques and highlighted microfluidization’s role in achieving consistently small, uniform particles; and Panagiotou et al. [14] demonstrated that high-shear microfluidizer processing effectively deagglomerates nanomaterials, yielding stable nanoscale dispersions. Collectively, these findings indicate that microfluidizer technology markedly enhances PLGANM stability and drug-loading capacity.

To enhance the stability of PLGANM using microfluidizer technology, several formulation and process factors must be considered. Firstly, the amount of PLGA is critical to ensure the desired drug loading capacity and appropriate degradation kinetics. The choice of surfactants or stabilizers that form the hydrophilic shell of the nano-micelle also plays a crucial role in stabilizing the formulation and preventing aggregation. This principle is supported by prior studies: Eskandari et al. [10] showed that optimizing surfactant content in a PEG-PLGA micelle formulation significantly reduces nanoparticle aggregation, and our group [15] demonstrated that incorporating a biopolymer stabilizer (levan) in a PLGANM improved the stability of curcumin-loaded nanoparticles. Additionally, the microfluidizer allows precise control over homogenization pressure, number of passes, and flow rate, which can influence the size, polydispersity, and stability of the nano-micelles. By systematically studying the impact of these factors on the CQAs of the nanoparticles, a QbD approach can be employed to optimize the formulation and process.

In this study, four factors were selected as critical variables. Two of these factors were the MFZ method variables, namely, pressure and the number of passes. One of the other two variables was the amount of Tween 80, which our group had identified as important in previous studies. In our previous studies, we noted that PLGANM is sterically stabilized by T80 after formation. Therefore, we chose the amount of T80 as a critical variable in this study [10,15]. In our previous studies, we noted that PLGANM is sterically stabilized by T80 after formation. Therefore, we chose the amount of T80 as a critical variable in this study. The fourth variable was selected as the amount of PLGA. Various studies systematically address the importance of PLGA amounts in the stability of PLGANM within broad analyses. These studies explore how PLGA concentration influences parameters such as particle size, aggregation kinetics, drug release, and degradation behavior, and consistently highlight the critical role of PLGA amount in nanoparticle stability across different synthesis methods and formulations [16,17,18].

Therefore, in the latter part of this work, various water-soluble natural antioxidant molecules were incorporated into the optimized PLGANM, and the preservation of their antioxidant activity (as an indicator of stability) was examined over time. We aimed to develop a preparation method that can be widely applied in research laboratories by combining MFZ and QbD techniques. We sought to produce consistently stable, one-dimensional PLGANMs for use in targeted cancer therapy using broadly applicable parameters. In this way, future researchers employing the same production method will not need to re-optimize these variables.

Conventional oil-in-water methods used to prepare poly(lactic-co-glycolic acid) nano-micelles (PLGANM) are increasingly applied because of their unique properties, yet they often suffer from poor physical stability. High-pressure microfluidizer (MFZ) technology can improve this stability, but full robustness cannot be achieved unless the critical formulation and process parameters are systematically optimized. In this context, the present study specifically integrates MFZ technology with a Quality by Design (QbD) approach to develop a reproducible PLGANM system. By optimizing key variables through design of experiments, we establish robust conditions for nano-micelle production, and validate the optimized MFZ-based PLGANM platform using antioxidant-loaded formulations, where long-term antioxidant stability serves as a functional readout of system performance and practical relevance.

## 2. Materials and Methods

### 2.1. Materials

PLGA, “Poly (lactic co-glycolic acid)” or “Poly (D, L-lactide-co-glycolide) lactide: glycolide” (50:50), mol wt. 30.000–60.00, Cat. No. P2191 was purchased from Sigma-Aldrich (St. Louis, MO, USA). Tween 80 (T80) Cat. No. 822187 and Acetone (HPLC grade) Cat. No. 100014 was obtained from Merck (Darmstadt, Germany). Water was purified and deionized using the Sartorius-Stedim Arium^®^ Water Purification System (Göttingen, Germany). Curcumin, Cat. No. C7727, Quercetin, Cat. No. Q4951, Genistein, Cat. No. G6649, Rosmarinic acid, Cat. No. R4033, Piperlongamine, Cat. No. SML0221, and 2,2-Diphenyl-1-picrylhydrazyl (DPPH) Cat. No. D9132 was purchased from Sigma-Aldrich.

### 2.2. Methods

#### 2.2.1. Creation of CCD Experimental Matrix with Quality by Design (QbD) Approach

We specifically aimed to develop X formulations using a QbD approach to understand the influences of formulation and process parameters on these nanoparticles’ CQAs and establish a design space.

The X drug delivery system was optimized using the response surface methodology. According to preliminary studies and literature review, PLGA amount, T80 amount, pressure level, and pass number have been selected as four important factors. Using a CCD, four factors were studied at 5 different levels (−α, −1, 0, +1, +α). The α value of 1.681 was chosen to maintain the rotatability and orthogonality of the design. A total of 60 experiments were carried out with 18 center points, 18 axial points, and 24 cube points (Table 1 and Table 2).

#### 2.2.2. Synthesis of PLGANM Using Microfluidizer (PMFZ)

**Scheme 1:**
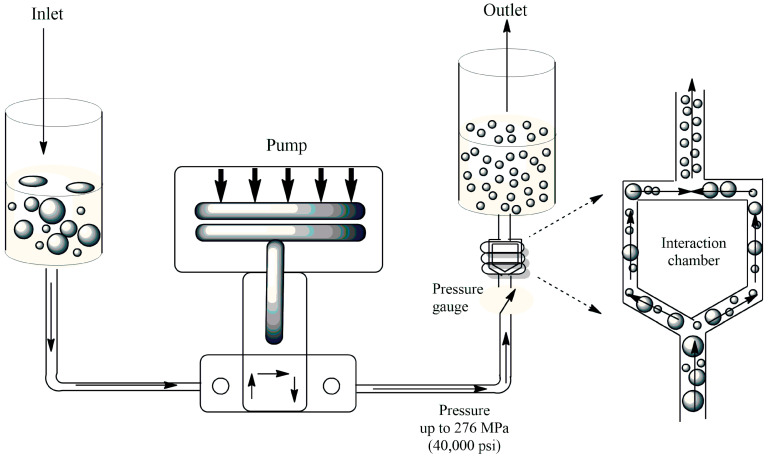
Schematic representation of the single-inlet high-pressure microfluidization system, the direction of water flow and the transformation of the formulation into stable one-dimensional nanoparticles during flow.

The oil-in-water (o/w) method was modified for the production of PLGANM using the microfluidizer method. Since the microfluidizer system is equipped with one inlet, the oil (organic) and aqueous phases need to be combined prior to introducing them to the MFZ system. Thus, the amounts of PLGA in mg, as given in Table 2, were dissolved in 12.5 mL of acetone and sonicated for 5 min (Bandelin Sonorex RK, Bandelin, Berlin, Germany). A 5% T80 stock solution was prepared in deionized water (d.w.) and was further diluted in 47.5 mL d.w. in the volumes specified in Table 2. The T80 solution was homogenized using a Tops SR 30-Rotor Stator Homogenizer, and the prepared PLGA solution in acetone was added to it while homogenization was continued. The o/w complex was further homogenized for 5 more minutes. The premixes, which were prepared with the values determined in the CCD, were transferred to the system from the inlet reservoir of the Microfluidizer device (STANSTED Pressure Cell- SPCH-EP, Harlow, Essex, UK), and each of them was transferred to the constant pressure pumping system. Scheme 1 illustrates the single-inlet high-pressure microfluidization system used for the preparation of the nanosuspension.

The system consists of a single-inlet high-pressure microfluidization setup. A coarse pre-emulsion containing the dispersed phase droplets is first loaded into the inlet reservoir and driven towards the pump. The high-pressure pump accelerates the suspension and delivers it at pressures up to 276 MPa (40,000 psi), which are monitored by a pressure gauge placed before the processing unit. Under this pressure, the fluid is forced through the microfluidic interaction chamber, where it experiences intense shear, impact, and extensional forces while flowing along the internal channels. These mechanical stresses progressively break down the large droplets into much smaller ones, and a homogeneous nanosuspension is finally collected at the outlet reservoir.

Following the number of passes determined in the CCD, each formulation was repeatedly passed through the device. After being processed in MFZ, the organic phase (acetone) was evaporated in RT on a magnetic stirrer for 12 h.

#### 2.2.3. Synthesis of PLGANM Using Classic (Manual) O/W Method (POW)

The classic o/w emulsification-evaporation method, which is followed by previous literature for the synthesis of PLGANM, was applied manually, and the obtained results in terms of particle size and stability were compared with those of the particles synthesized using MFZ technology. Since the pressure (K, psi) used in MFZ technology was not applicable in the classic method, only 10 formulations from the CCD experimental matrix were chosen to be prepared manually. The selected formulations consisted of all PLGA variables with the lowest and highest amounts of T80. The selected formulations are listed in Table 3.

To this end, the amounts of PLGA given in Table 3 as mg were dissolved in 12.5 mL of acetone and sonicated (Bandelin Sonorex RK, Bandelin, Berlin, Germany) for 5 min. A 5% stock solution of T80 in d.w. was prepared and was added to 47.5 mL d.w. in the volumes given in Table 3. The volume of d.w. and acetone was kept constant in all formulations. The aqueous media was placed on a magnetic stirrer, and the organic phase (PLGA in acetone) was added to the aqueous media in a very slow manner. Acetone was evaporated in RT for 12 h on the magnetic stirrer. The obtained formulations were further analyzed for particle size distribution, zeta potential, and stability, as it is described below.

#### 2.2.4. Evaluation of the Responses to the CCD Variables

Monodispersed NM, as the result of a successful production process, can be tracked by the single peaks obtained using the Dynamic Light Scattering (DLS) method. Destabilization of the colloidal system made of PLGANM results in obtaining multiple peaks in DLS studies, which mainly consist of aggregates with sizes in the micron range [19]. The change in the monodisperse behavior of NM is also detectable by the Poly Dispersity Index (PDI), calculated by Malvern Instruments, Zetasizer NanoZSP (Worcestershire, UK) [20]. Furthermore, the surface charge of NM shows variations in the formation of aggregates [21,22].

Thus, the stability of the produced PLGANM was studied based on changes in their size and surface charges by a quality-by-design approach. The stability of PMFZ was studied using Zetasizer. The particles dispersed in water were measured in a disposable cuvette at an angle of 173 degrees, accounting for a refractive index of 1.59 and absorption of 0.010. Zeta potentials were determined using the laser Doppler micro-electrophoresis method and the Helmholtz-Smoluchowski equation, utilizing a folded capillary zeta cell from Malvern Instruments Ltd. (Worcestershire, UK). The results were obtained as number, volume, and intensity of particle size distribution. Each measurement was repeated three times at a temperature of 25 °C. The measurements made on the 1st day were repeated on the 7th, 15th, 30th, and 60th days of particle production. The standard deviation (SD) of Z-Average, PDI, and zeta potential values was used as the responses of the CCD variables for 60 days [23].

#### 2.2.5. Comparison of the Stability of PMFZ and POW Particles Prior to the Optimization

Although the main purpose of the synthesis of 60 PMFZ particles using the CCD experimental matrix was to determine the parameters for the production of the optimized PMFZ (OPMFZ), these 60 particles were also used to compare the stability of particles produced using MFZ technology with their equivalents produced using classic method.

The size distribution measurements based on number, volume, and intensity were carried out at the end of the 1st, 7th, 15th, 30th, and 60th days of PMFZ and POW particle synthesis. The intensity distribution gives the amount of light scattered by the particles and is the most sensitive variable for determining minor changes in terms of size and shape [24]. In this study, we hypothesized that when a particle is completely stable, the change in particle size is zero, so the R^2^ value for the linear relationship between the intensity distribution of particle sizes and the recording days equals zero. The R^2^ value for PMFZ and POW was calculated to compare the stability of particles produced using MFZ and o/w methods. For this purpose, the average of R^2^ calculated for each formulation was accepted as a stability indicator value.

#### 2.2.6. Determination of the Optimized PMFZ (OPMFZ) Formulation Using QbD Approach

The equation that communicates between the studied factors and the answers is called a model. Here, the result can be expressed as a function based on experimental factors. There are three different models: linear model, interaction model, and quadratic model. In this study, the quadratic (second-order) model was used. Minitab 17 (Minitab Inc.; State College, PA, USA) software was used for experimental design and statistical analysis. The response will be predicted by three quadratic polynomial equations, each one representing one of the three levels (shown by i) of the categorical variable (i.e., Pass):

(1) $$Y_{i}\;=\;\beta_{0}\;+\;\beta_{1}X_{1}\;+\;\beta_{2}X_{2}\;+\;\beta_{12}X_{1}X_{2}\;+\;\beta_{11}X_{1}^{2}+\;\beta_{22}X_{2}^{2}$$

where Y is the predicted response (standard deviation of Z-average, PDI or zeta potential), X_1_ and X_2_ are the main effects of factors, X_1_X_2_ is the interaction effects of factors, X1 1 and X2 2 are the quadratic effects of factors, β_0_ is the constant, and β_1_ and β_2_ are the coefficients of the factors. Analysis of variance (ANOVA) will be performed to evaluate the effect of independent variables on the responses. After generating the polynomial equations regarding the factors and responses, formulation and process were optimized with respect to the standard deviations of average particle size (Y1), zeta potential (Y2), and PDI (Y3) of the X nano drug delivery system using the developed mathematical model to determine the levels of PLGA amount, T80 amount, and pressure level and Pass. Minimizing the standard deviations of average particle size (Y 1), zeta potential (Y 2), and PDI (Y 3) was adopted to determine the most stable formulation, one that shows less variety in different time intervals. Then, a design space was constructed using several contour and surface plots. The OPMFZ formulation was prepared and tested to evaluate the correlation between the predicted and the actual values of the responses.

#### 2.2.7. Determination of the Stability of OPMFZ, Drug Loading, and Comparison with POW

The OPMFZ formulation was synthesized using the experimental parameters determined by QbD studies. Particle size distribution in terms of number, volume, and intensity was measured along with the zeta potential of the optimized PMFZ (OPMFZ) using the DLS instrument as mentioned above. PDI and Z-average values calculated by the DLS instrument were also recorded. The measurements were repeated on the 7th, 15th, 30th, and 60th days of particle production.

Several natural compounds with different water solubilities were loaded onto the OPMFZ particles to compare the efficacy of the optimization process on the drug-carrying ability of PLGA. These molecules consist of curcumin (with 3 different concentrations, water solubility: 11 ng/mL [25,26]), quercetin (water solubility 10 μg/mL [27]), genistein (water solubility: 6.09 μg/mL [28]), rosmarinic acid (water solubility: 24,000 μg/mL [29]), piperlongumine (water solubility: barely soluble [30]). 3.32 mg, 5 mg, and 8.2 mg of curcumin (OPMFZLC, OPMFZMC, and OPMFZHC, respectively), 100 μL of genistein (OPMFZG), 4 mg of rosmarinic acid (OPMFZR), 8.5 mg of quercetin (OPMFZQ), and 1 mg of piperlongumine (OPMFZPi) were dissolved in acetone along with the optimized amounts of PLGA prior to homogenizing with the aqueous media containing the optimized amounts of T80. Particle size distribution parameters were also measured for 60 days for drug-carrying OPMFZ particles.

The experimental parameters for the OPMFZ consist of Pass Number, Pressure (psi) (Both for the MFZ technology), T80 (mL), and PLGA (mg). To evaluate the importance of using MFZ technology in producing PLGANM, the optimized values for T80 (mL) and PLGA (mg) were used to produce POW particles without passing through the MFZ instrument. Particle size distribution parameters were also measured for 60 days for POW particles. The same amounts of curcumin (POWLC, POWMC, and POWHC), rosmarinic acid (POWR), quercetin (POWQ), piperlongumine (POWPi), and genistein (POWQ) were dissolved in the organic phase along with PLGA and added to the aqueous media containing the optimized amounts of T80, homogenized, and the organic phase was evaporated at RT overnight. Particle size distribution parameters were also measured for 60 days for drug-carrying POW particles.

Based on this definition, the R^2^ values from the linear regression of intensity distribution versus storage time were calculated for PMFZ and POW and used to compare their particle stability.

#### 2.2.8. DPPH Radical Scavenging Activity of Drug-Loaded OPMFZ

The main goal of enhancing the stability of nano-drug delivery systems is to improve their biological activity. One of the most important properties of drug molecules, which is also detectable “on the bench” is antioxidant activity. The natural molecules chosen for studying the stability of OPMFZ were all well-known antioxidants. So, the ability of MFZ to enhance the stability of PLGA nanoparticles was investigated by following the antioxidant activity of the uploaded natural compounds for 60 days, along with the stability of the synthesized nanoparticles.

Slight adjustments were made to the 2,2-diphenyl-1-picrylhydrazyl (DPPH) assay, which is a stable free radical test to assess antioxidant activity. A stock solution of 0.0913 mM DPPH was prepared by dissolving 0.0036 g of DPPH in 100 mL of methanol. Each day, new working solutions were created by adequately diluting the stock solution with solvent [31]. Methanol was chosen as the solvent to make a monophasic environment with the aqueous OPMFZ samples. Also, since PLGA is soluble in methanol and the prepared PLGANM disassembles in methanol, this method provides the complete release of the loaded drug molecules to interact with DPPH. In each well of 96-well plates 160 μL of DPPH solution was added to 30 μL of the sample. An extra 10 μL of methanol was added to each well to ensure the disassembly of PLGANM. Drug-loaded OPMFZ was applied as produced, without dilution. The equivalent concentrations of the loaded molecules were dissolved in methanol and stored at RT along with OPMFZ formulations to compare the stability. An empty OPMFZ applied in the above-mentioned concentrations was used as control. DPPH absorbance was recorded at 517 nm at 1st and 60th days, and the inhibition of DPPH radical was calculated according to Equation (2) presented below.

(2) $$A=\frac{A_{Control}-A_{Sample}}{A_{Control}}\times 100$$

#### 2.2.9. Transmission Electron Microscopy (TEM) Analysis

TEM experiments were performed at an acceleration voltage of 80 kV using a JEOL JEM-2100 (Akishima, Tokyo, Japan) instrument. A copper grid measuring 0.037 mm was coated with PLGANM loaded with the high-dose Curcumin-carrying nanoparticles at a concentration of 1 mg/mL to prepare TEM samples. To produce a negative contrast on the grids, the samples were treated with a 2% (*w*/*v*) uranyl acetate solution in water (supplied by Electron Microscopy Sciences, Hatfield, PA, USA). The grids were then allowed to dry at room temperature after being cleaned with double-distilled water.

#### 2.2.10. Statistical Analysis

The average of the examined values was used to calculate the results, which were then statistically assessed using the non-parametric Tukey test for multiple comparisons and the ANOVA analysis of variance test. GraphPad Prism 5 was the computer program used for analysis. A *p*-value of less than 0.05 was deemed statistically acceptable. The acquired data are presented as mean ± S.E.M.

## 3. Results

### 3.1. Comparison of the Stability of PMFZ and POW Particles Prior to the Optimization

Number, volume, and intensity-based size distribution measurements were conducted on the 1st, 7th, 15th, 30th, and 60th days following the synthesis of PMFZ and POW particles. The intensity of the scattered light in DLS measurements was chosen as the determinative parameter in terms of the stability of the particle size distribution.

**Figure 1:**
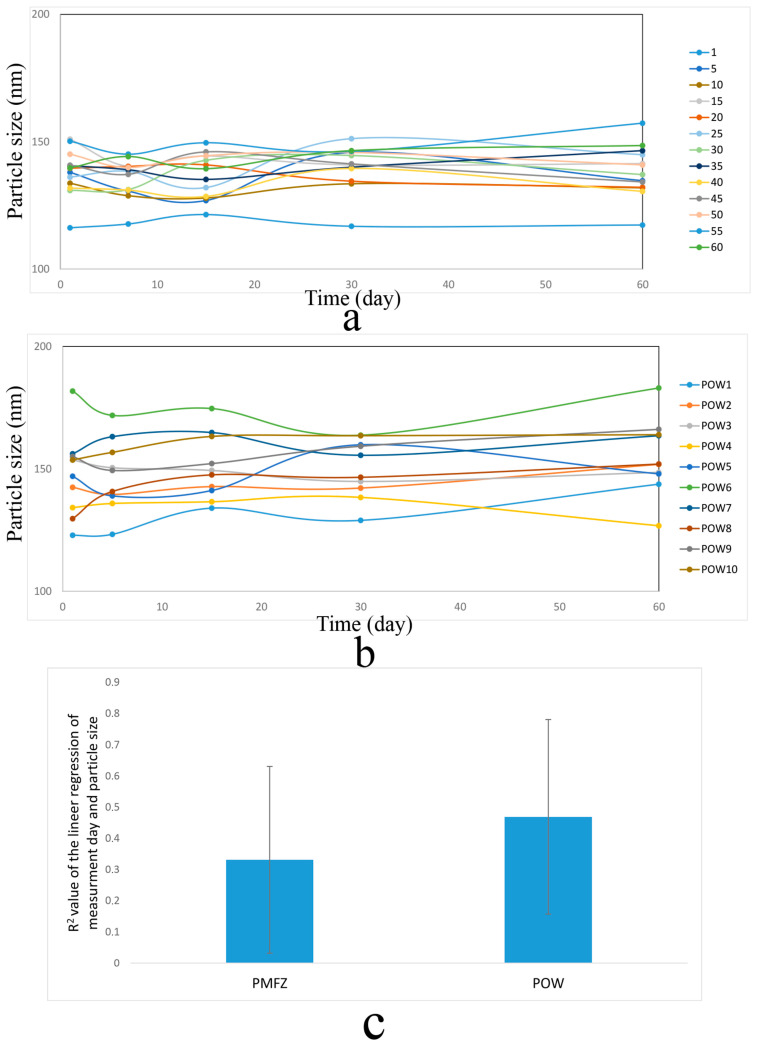
Particle size changes, prior to optimization by CCD shown for (**a**): selected PMFZ formulations and (**b**): POW formulations in 60 days reported by intensity distribution. 60 PMFZ particles were synthesized according to the CCD experimental matrix, along with their equivalents (POWs) produced using the classic method, and their stabilities were followed for 60 days. (**c**): The R^2^ value for the linear relationship between the intensity distribution of particle sizes and the recording days, calculated for 60 PMFZ and their equivalent POWs, indicating their statistically meaningless differences.

Figure 1 shows the changes in the sizes of PMFZ and POW particles for 60 days. The sizes of 13 randomly chosen particles from the prepared 60 PMFZ particles are shown in Figure 1a, and Figure 1b shows all synthesized POW particles. Particle size distribution of 60 PMFZ particles according to intensity, number, volume, PDI, Z-Average, and the zeta potential of particles measured for 60 days are listed in Supplementary Data S1.

Figure 1a shows the sizes of 13 randomly selected particles, and the average size of 60 PMFZ particles, which was calculated as 137.4 ± 4.3 nm (size distribution by intensity) at the end of the 60th day prior to optimization by QbD. This is where the average size distribution for POW particles was measured as 150.104 ± 6.1 nm. This is not a significant change in particle size (*p* > 0.05); nevertheless, it was important to note that the MFZ method produced particles with a size of less than 150 nm, and the majority of the particles were within a limited range of 130–150 nm. Conversely, the diameters of POW particles were distributed over a broader range of 122–181 nm.

In our study, we also posited that when a particle remains entirely stable, there will be no alterations in its size, and the R^2^ value for the linear correlation between the distribution of particle sizes and the duration of recording will be zero. We computed the average R^2^ values for PMFZ and POW to assess the stability of particles generated through the MFZ and o/w techniques for comparison. These R^2^ values for PMFZ and POW are shown in Figure 1c and were calculated as 0.33157 ± 0.2997, and 0.469178 ± 0.3119, prior to optimization by QbD, respectively. Although the R^2^ value for PMFZ is slightly smaller than that of POW, demonstrating the better stability of PMFZ particles over time, however, this difference was not found to be statistically meaningful (*p* > 0.05).

To ensure that the particle size of PMFZ was smaller than that of POW, as shown in Table 4, the average particle size was calculated based on all parameters, including size distribution by number and volume, the ODI value, and the Z-Average.

The average size of PMFZ particles was slightly smaller than that of POW particles in all measurement methods. However, this difference was not statistically significant (*p* > 0.05), indicating that the main advantage of the MFZ method lies in improved size distribution and stability rather than in a dramatic size reduction.

### 3.2. Experimental Design

#### 3.2.1. Design Model and Data Analysis

The CCD setup and obtained responses from a total of 60 experiments are presented in Table 4. The model fit was assessed. The lack of fit values was not significant for Z-average and Zeta potential (*p* = 0.12 and *p* = 0.37, respectively). However, for PDI, the lack of fit of the model (*p* = 0.002) was significant. Although the lack-of-fit was significant for PDI (*p* = 0.002), inspection of the residuals did not reveal any systematic trend over the design space, suggesting that the deviation is mainly related to the very low absolute PDI values and limited signal-to-noise ratio. For this reason, the PDI model was retained as a supportive response to penalize formulations with undesirably high PDI, rather than as a precise predictive tool.

The regression equations modeling the dependence of the standard deviation of Z-average, PDI, and zeta potential on formulation parameters (PLGA, Tween 80, pressure, and pass number) are summarized in Table 5.

Where Y1,1 and Y2,2 are particle sizes for different levels of PLGA molecular weight, while X1 and X2 are coded values for PLGA amount and PEG amount, respectively. The lack-of-fit value of the model was statistically insignificant (*p* = 2.85), which implies that the model fits well.

The detailed goodness-of-fit statistics (R^2^, adjusted R^2^, MS residual and standard deviation of residuals, S) for the second-order response surface models of SD of Z-average, SD of PDI, and SD of zeta potential at each pass level are summarized in Supplementary Data S2.

Table 6 presents the standard deviation values for the CCD experimental formulations, reflecting the variability of the measured responses.

#### 3.2.2. Determination of the Coded Variables for Production of the OPMFZ

Optimum conditions were determined by Minitab 17 software based on the results of the CCD study. First, the average values of Z-average, PDI, and zeta potential were examined to confirm that all formulations remained within predefined acceptable ranges (nanometric size, low PDI, and sufficiently high absolute zeta potential; see Table 4). Since no practically relevant trend in the mean values was observed within the design space, the numerical optimization step was then focused on minimizing the standard deviations of these parameters over 60 days, which were used as indicators of long-term physical stability. Coded variables for optimized formulation are presented in Table 7.

The composite desirability of the optimization model was 0.954, which indicated a very good solution. Three-dimensional surface plots and contour plots for standard deviations of Z-average, PDI, and Zeta Potentials as a function of PLGA amount, T80, pressure, and pass are presented in Supplementary Data S3 and Figures 5–7.

The response surface models used to optimize nanoparticle formulations are presented as Supplementary Data Figures S1–S3. These plots highlight the interaction effects between formulation variables, demonstrating that stability is maximized within specific ranges of PLGA and Tween 80 concentrations. For instance, as observed in Supplementary Data Figure S1, excessive PLGA concentrations lead to increased particle size variation, whereas a moderate balance with Tween 80 results in smaller and more stable particles. The trends observed in these figures were further validated using statistical analysis, confirming the significance of these relationships (ANOVA, *p* < 0.05). These findings directly guided the selection of optimized formulation conditions. It should be noted that these figures are intended to illustrate general trends rather than serve as direct statistical proof. The conclusions drawn in the study are supported by statistical analysis elsewhere, ensuring that the observed trends are validated with significance testing.

**Figure 2:**
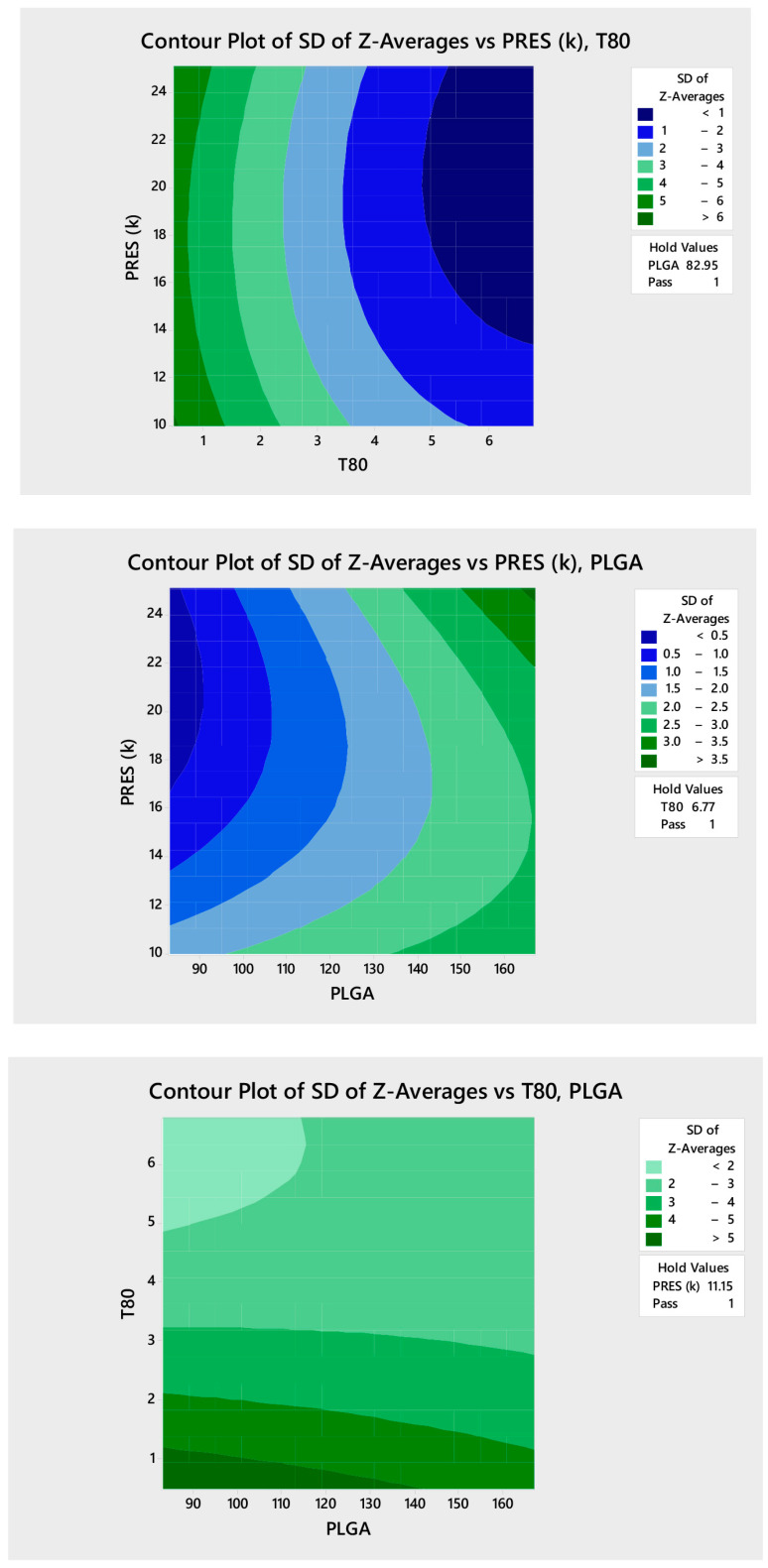
Contour plot showing the standard deviation of Z-average as a function of PLGA amount, Tween 80 concentration, pressure, and pass number. The contour lines indicate the regions of minimal variability, highlighting the conditions that lead to stable nanoparticle formulations with consistent size distribution.

**Figure 3:**
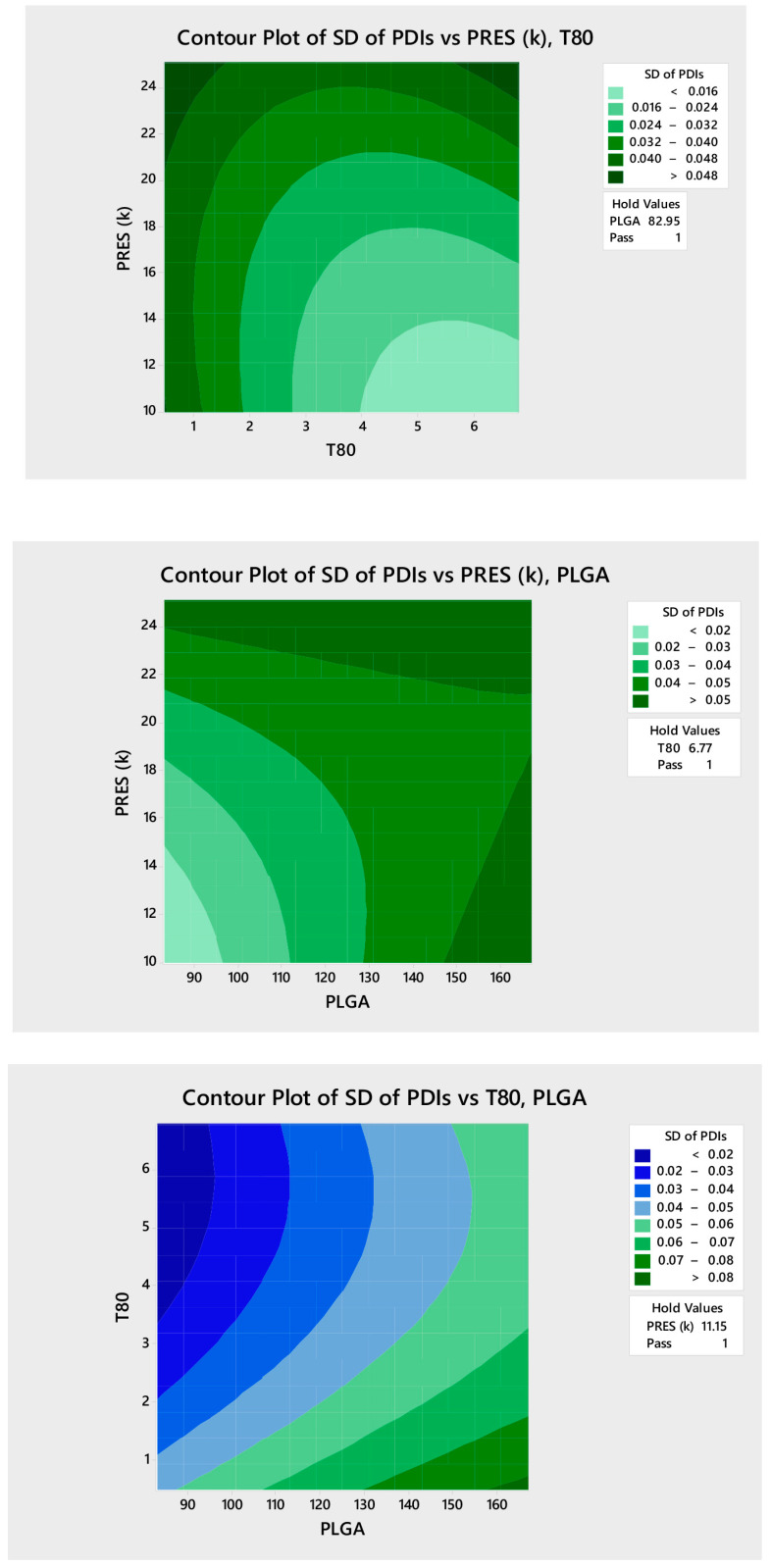
Contour plot depicting the standard deviation of the polydispersity index (PDI) in response to varying levels of PLGA, Tween 80, pressure, and pass number. The optimal region, where PDI remains lowest, is highlighted to indicate the formulation conditions that produce monodisperse nanoparticles.

**Figure 4:**
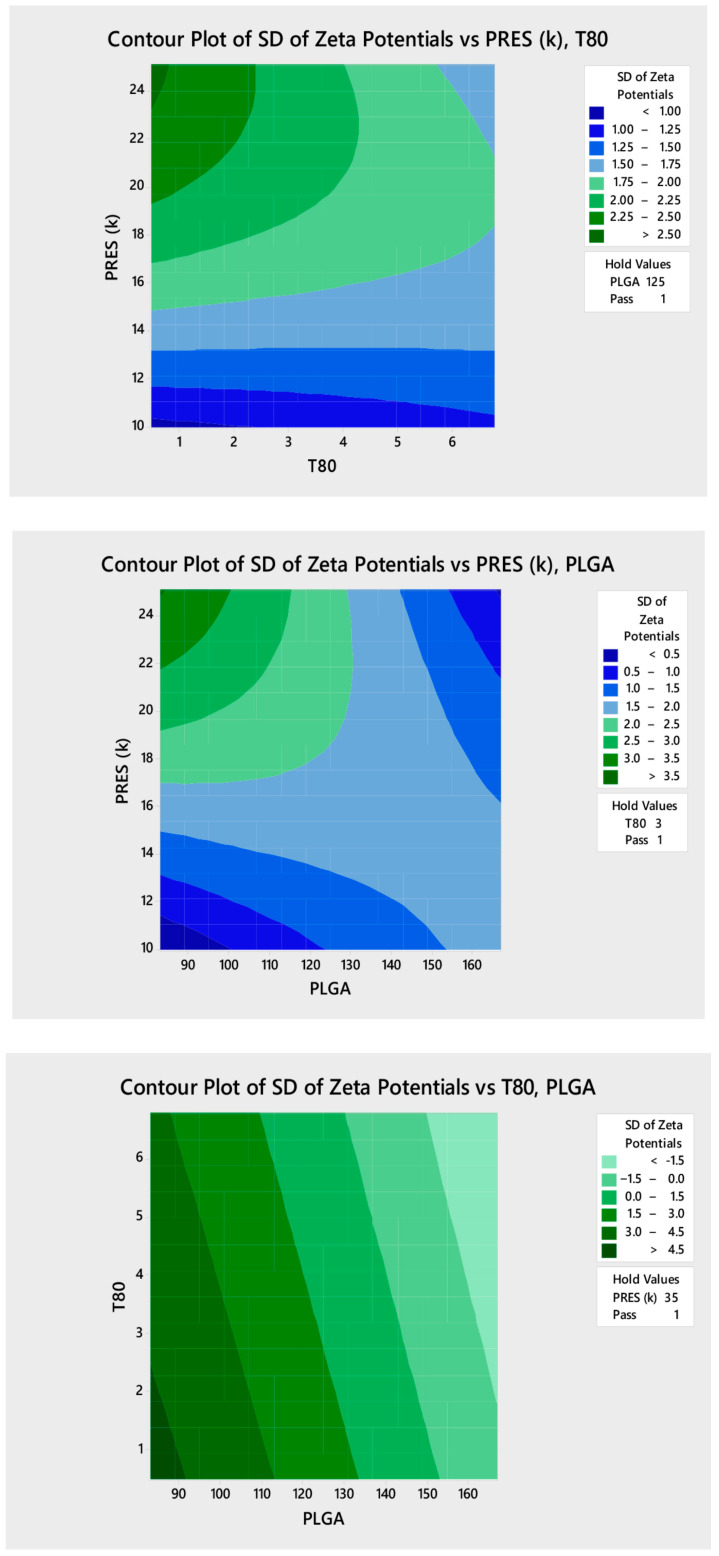
Contour plot illustrating the impact of formulation variables on the standard deviation of zeta potential. The graph identifies the conditions under which nanoparticles maintain stable surface charge properties, ensuring colloidal stability over time.

Figure 2, Figure 3 and Figure 4 present two-dimensional contour plots that complement the three-dimensional surface plots in Supplementary Data S3. These 2D plots provide a clearer view of the parameter interactions by mapping the regions where variability in nanoparticle characteristics (size, PDI, and zeta potential) is minimized.

Unlike the 3D surface plots, which provide an overview of parameter trends, the contour plots in Figure 2, Figure 3 and Figure 4 allow for a more precise identification of formulation conditions that yield the most stable nanoparticles. The darker or more concentrated contour regions indicate areas where particle stability is optimized, supporting the selection of the optimized PMFZ formulation with minimal size variation, controlled PDI, and stable zeta potential.

Together, these figures reinforce the conclusions drawn from the Quality by Design (QbD) optimization process, demonstrating the statistical relationships between formulation variables and nanoparticle stability.

#### 3.2.3. Evaluating the OPMFZ Formulation

The optimized formulation was prepared in triplicate to evaluate the model accuracy for the optimum conditions. Predicted and experimental responses for optimized variables are presented in Table 8. Observed experimental values were in close agreement with the predicted values. Coordination between the results indicated the significance and validity of the model.

### 3.3. Determination of the Stability of OPMFZ, Drug Loading, and Comparison with POW

**Figure 5:**
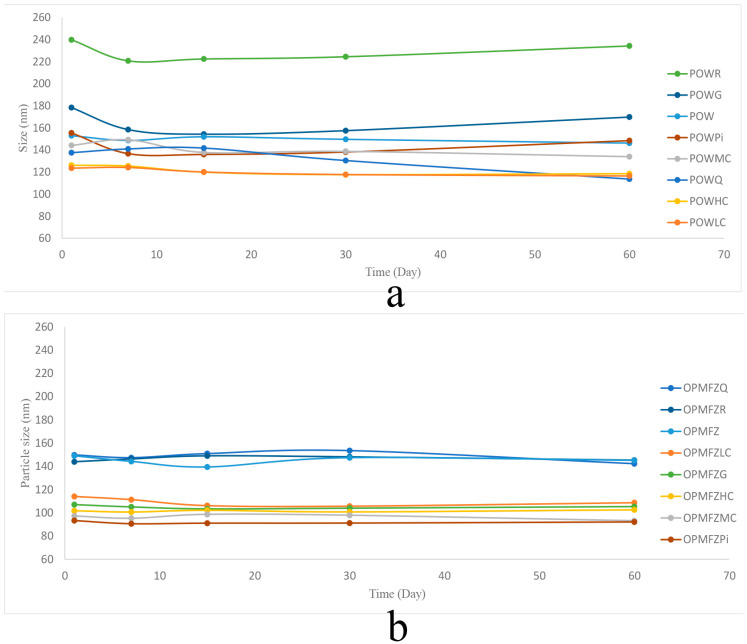
The intensity of the particle size distribution of drug-loaded (**a**) POW and (**b**) OPMFZ, showing the smaller sizes of drug-loaded OPMFZ compared to that of POW.

The drug-carrying capability of PLGA was assessed by incorporating various naturally occurring bioactive compounds with distinct water solubilities into the OPMFZ particles, aiming to evaluate the effectiveness of the optimization process. Figure 5a shows the particle size distribution of drug-loaded manually prepared optimized particles for 60 days. While all particles showed an intensity distribution above 120 nm, the Resveratrol-loaded POW (POWR) showed a significantly larger size. As seen in Figure 5b, the sizes of drug-loaded OPMFZ particles are divided into two groups with statistically meaningful size differences (*p* < 0.05). The particles with larger sizes are the ones that carry the hydrophilic drugs, namely OPMFZQ and OPMFZR. The size of the empty OPMFZ is also large. The sizes of PLGA nanoparticles carrying hydrophobic drug molecules consisting of all concentrations of curcumin (OPMFZLC, OPMFZMC, and OPMFZ HC), piperlongumine (OPMFZPi), and genistein (OPMFZG) demonstrated a smaller size during the 60 days of stability studies.

To assess the stability of the PLGANM over time, we analyzed the intensity-weighted particle size distribution obtained from Dynamic Light Scattering (DLS) measurements at predetermined time points (1, 7, 15, 30, and 60 days). The intensity-based size distribution was chosen because it is the most sensitive to detecting aggregation or instability, as larger particles scatter light and contribute disproportionately to the measurement.

**Figure 6:**
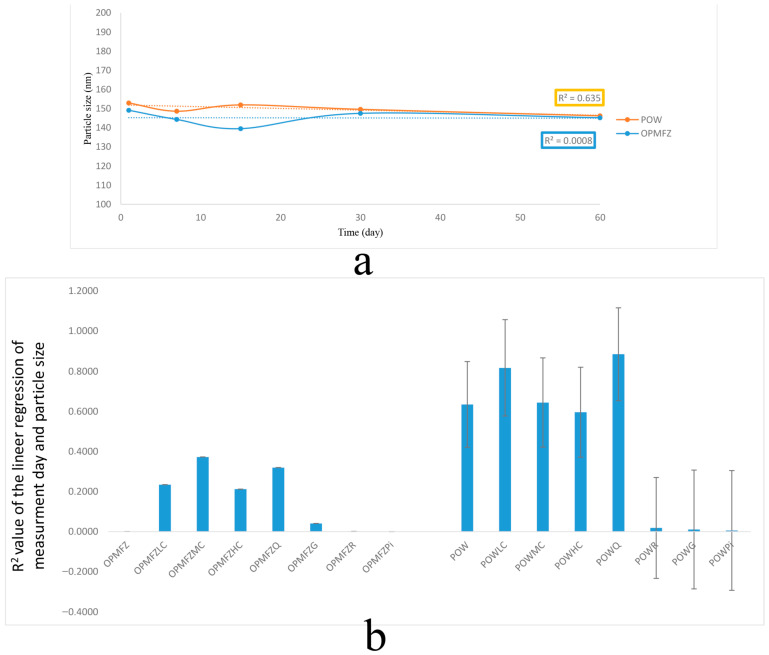
(**a**) Particle size changes in OPMFZ and its equivalent produced using classic method in 60 days reported by intensity distribution with corresponding R^2^ values showing the higher stability of OPMFZ with lower R^2^ values and (**b**) Particle size changes in drug-loaded OPMFZ and their classically prepared equivalents in 60 days reported by intensity distribution with corresponding R^2^ values showing the higher stability and smaller size of OPMFZ with smaller standard deviation values. Therefore, the microfluidizer-based method can be considered superior in terms of long-term size stability when compared with the manually prepared POW formulation.

To quantify stability trends, a linear regression model was applied to examine the relationship between particle size (intensity-based) and storage duration. The coefficient of determination (R^2^) was calculated to evaluate the degree of size variation over time, with lower R^2^ values indicating greater stability (i.e., minimal size fluctuations) and higher R^2^ values suggesting instability. The optimized PMFZ formulation exhibited a significantly lower R^2^ (0.0008 ± 0.0002) compared to POW, produced using classic method (0.635 ± 0.21) (*p* < 0.05) (Figure 6a), demonstrating superior stability of the microfluidizer-based approach while it is combined with QbD.

This MFZ-based, QbD-guided approach enabled the reproducible production of stable PLGA nano-micelles suitable for drug loading. In contrast, the conventional oil-in-water method yielded particles with broader size distributions and less favorable stability profiles over time. Figure 6b shows the better stability and smaller sizes of drug-loaded OPMZ particles compared to their drug-loaded and manually prepared POW particles.

### 3.4. DPPH Radical Scavenging Activity of Drug-Loaded OPMFZ

The most important indicator of the improved stability of a nanocarrier, in addition to improving its physicochemical properties, is the maintenance of the activity of the carried drug molecules for a longer time. Since the chosen drug molecules with different polarities were all-natural antioxidant molecules, their DPPH radical scavenging activity was analyzed for 60 days as a marker of stability enhancement. OPMFZ was disassembled using methanol before recording the DPPH absorbance to achieve the complete release of the loaded drugs.

**Figure 7:**
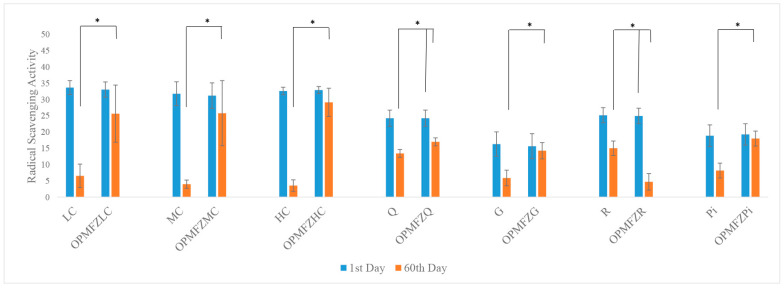
DPPH radical scavenging activity of drug-loaded OPMFZ and the equivalent bare drug molecules. *: *p* < 0.05.

Figure 7 shows the DPPH radical scavenging activity of 7 drug-loaded OPMFZ formulations. While the DPPH radical scavenging activity of bare molecules was equal to their equivalent OPMFZ formulations on the first day of analysis, all OPMFZ formulations except OPMFZR showed higher DPPH scavenging activity at the end of the 60th day. The DPPH radical scavenging activity of all OPMFZ formulations did not show a statistically meaningful decrease (*p* < 0.05) at the end of the 60th day compared to that of the 1st day, except for OPMFZG and OPMFZR. The activity of OPMFZG was reduced compared to the 1st day (still higher than the activity of bare G on the 60th day), and the activity of OPMFZR was reduced compared to R at the end of the 60th day.

### 3.5. TEM Images of Curcumin-Carrying and Empty PLGANMs

**Figure 8:**
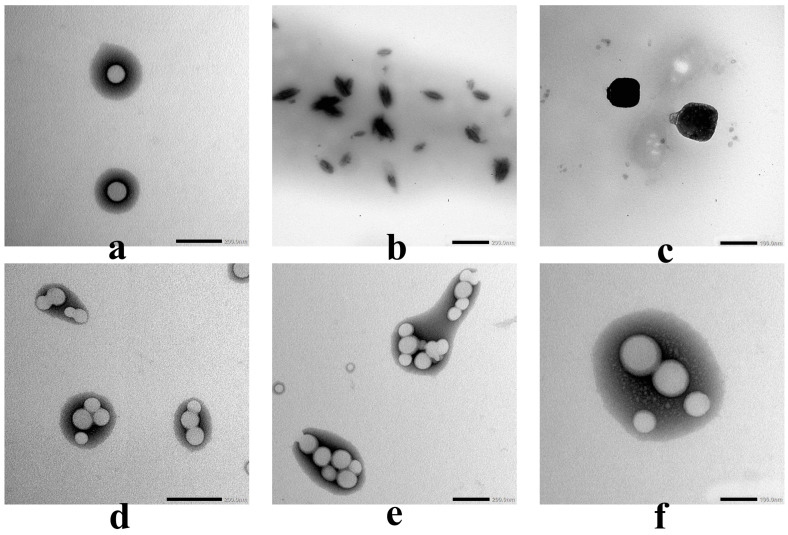
The TEM images of (**a**): POWHC right after production, (**b**): after 60 days with low magnification (scale bar = 200 nm), and (**c**): after 60 days with high magnification (scale bar = 100 nm). (**d**): OPMFZHC right after production, (**e**): after 60 days with low magnification (scale bar = 200 nm), and (**f**): after 60 days with high magnification (scale bar = 100 nm).

Figure 8 shows the TEM images of POWHC and OPMFZHC right after production and after 60 days. While the change in the morphology of PLGANM produced by the o/w method is seen, it is clearly shown that OPMFZ causes the shape of PLGANM to remain constant for 60 days.

## 4. Discussion

The unstable nature of colloidal carriers such as PLGANMs is one of their primary drawbacks. During storage, they frequently form agglomerates in aqueous media. This is because of the system’s large surface area and its resulting thermodynamic instability, which encourages the colloidal particles to agglomerate. The practical use of nanoparticulate carrier systems in medicine is severely limited by their intrinsic instability.

The challenges and intricacies of preparation techniques have restricted the production of PLGANM. The production of PLGANM using conventional methods has several drawbacks, such as high production costs; the need for labor-intensive procedures, prolonged mechanical mixing, and the difficulty of producing stable particles in large quantities with ease and consistency. This field is still very unexplored, and the biggest obstacles still have to be overcome. These include scaling up the process for commercialization, reproducibility of the approach, and careful research into preparation techniques that result in high drug entrapment efficiency [32].

Microfluidizer systems have previously been used in the production of PLGANM in several studies [11,12,13,14,33]. The MFZ procedure was shown to be effective in achieving a higher degree of emulsion stability in earlier studies on the development of submicron emulsion systems [34].

PLGA has been the subject of numerous studies employing the double emulsion solvent evaporation method (POW). Numerous variables have been taken into account in these works, including the kind of solvents and polymer, the concentration of the polymer, the molecular weight of the polymer, the kind and concentration of surfactants, the homogenization technique, the duration and intensity, the phase ratio, and so on. The properties of micro- or nanoparticles can be influenced by all of these variables, contingent on the circumstances of the experiment. Modifying each of these variables can have an impact on several aspects, such as drug loading and release pattern, zeta potential, polydispersity index, encapsulation efficiency, and particle size and distribution. It is clear that a higher surfactant content (>3%) results in a more stable emulsion and, ultimately, can yield smaller particles. It has been demonstrated that the kind and concentration of surfactants, the concentration of polymers, and the speed of homogenization are all closely correlated with particle size [35,36].

The QbD method has also been employed in obtaining stable PLGANM, considering several variables in the production process [37,38]. According to the best of our knowledge, this is the first report on the optimization of producing PLGANM using both MFZ and QbD technologies, while the obtained results were also compared with those of traditional oil-in-water emulsification methods. To compare the efficacy of the MFZ method, the equivalent amounts of PLGA and T80 were used to manually produce PLGANM (POW) without employing the high pressure and the pass number. In the MFZ method, 60 particles were produced based on the variables defined by CCD. Amounts of PLGA and 5% T80, pass number, and MFZ pressure were chosen as the variables of the formulation.

In addition to the conventional evaluation of particle size and PDI changes over time, R^2^ values from the regression of intensity-based size distribution versus storage time were calculated as an auxiliary descriptor of time-dependent drift. This unconventional metric was not used as a stand-alone stability criterion but rather as a complementary summary of whether any systematic trend beyond experimental variability could be detected. Based on this perception, the mentioned R^2^ value was calculated for the produced PMFZ and POW particles based on the measurements carried out for 60 days. While performing this analysis, the particle size was taken in the form of the “intensity distribution” due to its sensitivity to variations [39].

Before the optimization of particles using the CCD, setup and obtaining responses, the standard deviation of the differences between the above-mentioned R^2^ value for PMFZ and POW was not meaningful. However, it was noteworthy that the size of all particles produced using the MFZ method was below 150 nm, and the sizes of most particles were in a narrow range between 130 and 150 nm. On the contrary, the sizes of POW particles were distributed in a wider range from 122 to 181 nm (Figure 1). Calculation of the average particle sizes of 60 PMFZ and 10 POW particles based on intensity, number, and volume distribution (Table 4) also showed the smaller sizes of all PMFZ particles during the measurement period compared to those of POW. However, the particle size was not directly proportional to any of the variable values. Neither high (or low) amounts of PLGA or T80 nor a certain pressure value or pass number caused the production of smaller particles. Accordingly, the responses to the variables of the CCD experimental matrix resulted in obtaining values shown in Table 7.

The OPMFZ was obtained according to these values, and the particle size measurement was continued for 60 days. Table 8 presents the observed and predicted values of the optimal PLGA nanoparticle formulation based on the desirability function. The observed values for PLGA (82.96 mg), T80 (6.78 mL), and pressure (11,000 psi) closely match the predicted values of 82.9552 mg, 5.31316 mL, and 16.3533 psi, respectively, indicating a high degree of accuracy in the optimization model. The pass number was accurately predicted at 1, further validating the robustness of the Quality by Design (QbD) approach used in this study. The minimal residuals between observed and predicted values demonstrate the model’s effectiveness in optimizing the critical process parameters for achieving stable and effective PLGA nanoparticles. The residuals calculated were 0.0048 for PLGA, 1.46684 for T80, 10,983.6467 for pressure, and 0 for the pass number. The identification of optimal parameters at the edges of the studied range suggests that the true optimal conditions may reside at or beyond the current experimental boundaries. This outcome highlights the need for further investigation into these boundary regions to fully understand the behavior of the system under study.

To quantitatively assess nanoparticle stability, we analyzed the linear regression of particle size (from intensity-based DLS measurements) over time and calculated R^2^ as an indicator of size variability. If R^2^ is considered as the dependent variable, its interpretation shifts from being a general model fit measure to a stability indicator, where lower values suggest minimal particle size fluctuations and greater stability.

The optimized PMFZ formulation showed an R^2^ of 0.0008 ± 0.0002, indicating that particle size remained virtually unchanged throughout the study period. Conversely, the POW formulation produced using the classic method exhibited a significantly higher R^2^ of 0.635 ± 0.21, reflecting greater instability due to changes in particle size over time.

Thus, in the context of nanoparticle formulations, treating R^2^ as a dependent variable allows us to quantify the impact of formulation parameters on size stability, reinforcing the superiority of the microfluidizer-based method. To demonstrate that successful stability results can be achieved not only with empty particles but also with drug-loaded particles, natural compounds with various water solubilities were loaded into OPMFZ. The selected natural compounds were also all known antioxidants. The contribution of the antioxidant compounds also prepared the equivalent POW formulations to OPMFZ ratios. Figure 5 shows the above-mentioned R^2^ values for drug-loaded OPMFZ and POW. As shown in Figure 5a, the drug-loaded POW particles exhibited a distribution over a very wide range, and their sizes did not decrease compared to the empty POW particles before optimization. However, as shown in Figure 5b, drug-loaded OPMFZ particles showed not only sizes smaller than PMFZ but also their distributions were collected in a very narrow area from 93 to 149 nm. One more important result obtained from loading drug molecules into OPMFZ was related to the hydrophilicity of these drug molecules. OPMFZQ and OPMFZR, carrying quercetin and rosmarinic acid, respectively, showed the highest particle sizes. Quercetin and resveratrol were the most hydrophilic compounds in this research. This shows better incorporation of hydrophobic compounds with PLGANM by the MFZ method, resulting in obtaining smaller particles. Figure 6a,b also reveal the higher stability of OPMFZ formulations compared to their POW equivalents. The size variations in the drug-loaded POW formulations were statistically meaningless.

In our interpretation of the design space, primary emphasis was placed on Z-average and zeta potential, for which the quadratic models showed non-significant lack-of-fit. In contrast, the PDI model was used qualitatively to ensure that optimized conditions did not lead to increased polydispersity, acknowledging its limited predictive accuracy due to the significant lack-of-fit.

The DPPH radical scavenging activity of the bare natural antioxidants was compared to the ones encapsulated in OPMFZ for 60 days. As shown in Table 7, the activity of the encapsulated antioxidants was higher than their bare equivalents at the end of 60 days for all compounds except for OPMFZR. On the other hand, the comparison of the activity of the encapsulated antioxidants at the first and 60th days showed their significant stability during this period, except for OPMFZR and OPMFZQ. As mentioned above, these two formulations carry the compounds with the highest water solubility. These findings suggest that highly hydrophilic compounds may be located preferentially in the outer shell of PLGA nano-micelles, leading to faster activity loss. To address this limitation, future formulations could incorporate additional stabilizers or alternative encapsulation strategies for hydrophilic antioxidants.

## 5. Conclusions

This study integrated MFZ technology with a QbD framework to develop a reproducible PLGA nano-micelle (PLGANM) system. Using a central composite design, we identified an optimized MFZ formulation that produced nano-sized particles with narrow size distribution and low polydispersity, and showed significantly lower variability in Z-average, PDI, and zeta potential over 60 days than the manually prepared POW suspension.

The optimized MFZ formulation maintained its physicochemical properties during storage and preserved antioxidant activity, demonstrating that the process conditions obtained by QbD translate into functionally stable nano-micelles. TEM images further confirmed that MFZ processing yields discrete, well-dispersed particles, whereas POW leads to large aggregates, highlighting the superiority of the microfluidic route for long-term physical stability.

Our work, therefore, provides a practical roadmap for combining MFZ and QbD to design stable, scalable PLGANM platforms for parenteral delivery. Future studies will extend this approach to direct comparisons of encapsulation efficiency and biological activity between POW and MFZ products, refine stability metrics beyond exploratory R^2^ analysis, and explore a broader range of therapeutic cargos and process scales.

## Figures and Tables

**Table 1:** The variables chosen in the CCD.

Factor	Level				
	−α	−1	0	+1	+α
X1: PLGA amounts (mg)	83	100	125	150	167
X2: T80 amounts (mL)	0.5	1.8	3.6	5.5	6.8
X3: Pressure level in MFZ (psi × 1000)	10	13	18	22	25
X4: Pass number in MFZ	1		3		5

**Table 2:** The CCD experimental matrix.

Formulation Number	PLGA (mg)	T80 (mL)	Press. psi × 1000	Pass	Formulation Number	PLGA (mg)	T80 (mL)	Press. Psi × 1000	Pass
1	100	1.8	22	1	31	125	6.8	18	1
2	125	3.6	25	5	32	100	1.8	13	3
3	125	3.6	18	1	33	100	5.5	13	5
4	150	5.5	22	1	34	125	3.6	18	3
5	125	6.8	18	3	35	150	5.5	13	5
6	83	3.6	18	1	36	150	1.8	22	3
7	150	5.5	13	1	37	150	1.8	22	5
8	100	1.8	13	5	38	125	3.6	25	3
9	125	0.5	18	3	39	125	3.6	18	3
10	100	5.5	13	1	40	125	3.6	18	5
11	150	5.5	13	3	41	100	5.5	22	3
12	125	3.6	18	1	42	125	3.6	18	5
13	167	3.6	18	1	43	150	1.8	13	3
14	150	1.8	13	5	44	100	5.5	13	3
15	150	1.8	13	1	45	125	3.6	18	1
16	100	1.8	13	1	46	125	3.6	25	1
17	83	3.6	18	3	47	125	0.5	18	1
18	125	3.6	18	5	48	150	5.5	22	5
19	125	3.6	18	1	49	125	0.5	18	5
20	125	3.6	18	1	50	125	3.6	18	5
21	100	1.8	22	3	51	125	3.6	18	3
22	125	3.6	10	3	52	100	5.5	22	5
23	125	3.6	10	5	53	125	3.6	18	3
24	100	5.5	22	1	54	125	6.8	18	5
25	150	1.8	22	1	55	167	3.6	18	5
26	125	3.6	18	3	56	100	1.8	22	5
27	125	3.6	18	3	57	125	3.6	10	1
28	167	3.6	18	3	58	125	3.6	18	5
29	83	3.6	18	5	59	150	5.5	22	3
30	125	3.6	18	5	60	125	3.6	18	1

**Table 3:** The formulations prepared using the classic method based on the CCD experimental matrix.

Formulation Number	PLGA (mg)	T80 (mL)
POW1	83	0.5
POW2	83	6.8
POW3	100	0.5
POW4	100	6.8
POW5	125	0.5
POW6	125	6.8
POW7	150	0.5
POW8	150	6.8
POW9	167	0.5
POW10	167	6.8

**Table 4:** The average particle size of 60 PMFZ and 10 POW particles prior to optimization by QbD based on intensity, number, and volume distribution, along with the PDI value and the Z-Average during the 60 days of measurements.

Size Distribution Measurement Methods	Formulation Method	Measurement Duration				
		1 ± STDV	7 ± STDV	15 ± STDV	30 ± STDV	60 ± STDV
Intensity	PMFZ	138.46 ± 35	137.67 ± 25	136.05 ±28	138.72 ± 34	136.18 ± 35
	POW	147.68 ± 24	147.04 ± 35	150.67 ± 28	150.33 ± 31	154.8 ± 33
Number	PMFZ	100.85 ± 22	95.210 ± 29	99.315 ± 20	99.08 ± 30	95.62 ± 33
	POW	98.586 ± 34	105.04 ± 32	97.84 ± 25	106.92 ± 29	100.82 ± 37
Volume	PMFZ	125.86 ± 21	122.62 ± 31	123.41 ± 27	125.27 ± 31	121.38 ± 28
	POW	132.63 ± 22	134.78 ± 25	134.47 ± 33	138.28 ± 21	139.2 ± 34
PDI	PMFZ	0.08185	0.082183	0.079167	0.073017	0.088017
	POW	0.162	0.0806	0.1093	0.1057	0.1045
Z-Average	PMFZ	128.25	126.29	126.40	128.1467	125.4967
	POW	135.97	135.94	135.99	137.75	138.82

**Table 5:** Regression model equations describing the dependence of nanoparticle stability parameters (particle size, polydispersity index, and zeta potential) on formulation variables (PLGA amount, Tween 80 concentration, pressure, and pass number). The equations include linear, quadratic, and interaction terms.

Response Variable	Pass Number	Model Equation
Z-average (SDVzAVE)	1	SDVzAVE = 12.47 − 0.0303 PLGA − 1.670 T80 − 0.564 PRES (k) − 0.000027 PLGA × PLGA + 0.0994 T80 × T80 + 0.01202 PRES (k) × PRES (k) + 0.00484 PLGA × T80 + 0.00170 PLGA × PRES (k) − 0.0136 T80 × PRES (k)
	3	SDVzAVE = 12.84 − 0.0556 PLGA − 1.229 T80 − 0.479 PRES (k) − 0.000027 PLGA × PLGA + 0.0994 T80 × T80 + 0.01202 PRES (k) × PRES (k) + 0.00484 PLGA × T80 + 0.00170 PLGA × PRES (k) − 0.0136 T80 × PRES (k)
	5	SDVzAVE = 7.55 − 0.0258 PLGA − 0.859 T80 − 0.489 PRES (k) − 0.000027 PLGA × PLGA + 0.0994 T80 × T80 + 0.01202 PRES (k) × PRES (k) + 0.00484 PLGA × T80 + 0.00170 PLGA × PRES (k) − 0.0136 T80 × PRES (k)
PDI (SDVPDI)	1	SDVPDI = −0.0165 + 0.001273 PLGA − 0.02022 T80 + 0.00015 PRES (k) − 0.000002 PLGA × PLGA + 0.001181 T80 × T80 + 0.000098 PRES (k) × PRES (k) + 0.000019 PLGA × T80 − 0.000041 PLGA × PRES (k) + 0.000406 T80 × PRES (k)
	3	SDVPDI = −0.0107 + 0.000997 PLGA − 0.01809 T80 + 0.00134 PRES (k) − 0.000002 PLGA × PLGA + 0.001181 T80 × T80 + 0.000098 PRES (k) × PRES (k) + 0.000019 PLGA × T80 − 0.000041 PLGA × PRES (k) + 0.000406 T80 × PRES (k)
	5	SDVPDI = −0.0646 + 0.001246 PLGA − 0.01482 T80 + 0.00158 PRES (k) − 0.000002 PLGA × PLGA + 0.001181 T80 × T80 + 0.000098 PRES (k) × PRES (k) + 0.000019 PLGA × T80 − 0.000041 PLGA × PRES (k) + 0.000406 T80 × PRES (k)
Zeta Potential (SDVZETA)	1	SDVZETA = −10.19 + 0.0794 PLGA + 0.158 T80 + 0.786 PRES (k) − 0.000092 PLGA × PLGA + 0.0015 T80 × T80 − 0.00593 PRES (k) × PRES (k) − 0.00001 PLGA × T80 − 0.00372 PLGA × PRES (k) − 0.0128 T80 × PRES (k)
	3	SDVZETA = −8.75 + 0.0748 PLGA + 0.261 T80 + 0.683 PRES (k) − 0.000092 PLGA × PLGA + 0.0015 T80 × T80 − 0.00593 PRES (k) × PRES (k) − 0.00001 PLGA × T80 − 0.00372 PLGA × PRES (k) − 0.0128 T80 × PRES (k)
	5	SDVZETA = −11.50 + 0.0844 PLGA + 0.470 T80 + 0.751 PRES (k) − 0.000092 PLGA × PLGA + 0.0015 T80 × T80 − 0.00593 PRES (k) × PRES (k) − 0.00001 PLGA × T80

**Table 6:** Standard deviation values obtained for the CCD experimental formulations (responses represent variability measurements).

Formulation Number	Responses (Standard Deviations)		
	SD * Z-Ave	SD * PDI	SD * Zeta
1	2.86	0.039	4.86
2	2.5	0.043	0.99
3	1.64	0.022	1.87
4	1.53	0.043	0.46
5	1.94	0.035	0.69
6	1.36	0.032	0.65
7	3.31	0.035	1.87
8	2.15	0.023	1.24
9	3.66	0.061	0.38
10	1.25	0.033	1.4
11	2.59	0.053	1.43
12	0.73	0.021	0.74
13	1.6	0.043	1.3
14	1.8	0.032	0.99
15	3.99	0.072	1.26
16	5.47	0.039	0.86
17	1.39	0.022	0.98
18	1.96	0.031	1.51
19	2.18	0.039	0.94
20	2.29	0.045	1.76
21	7.82	0.062	0.64
22	2.11	0.044	1.96
23	1.82	0.018	0.65
24	1.55	0.033	2.31
25	5.43	0.049	1.78
26	3.84	0.042	0.5
27	0.93	0.034	0.71
28	1.72	0.021	0.66
29	1.96	0.023	0.9
30	4.16	0.032	1.39
31	1.6	0.045	1.83
32	2.87	0.034	2.8
33	3.37	0.023	2.11
34	1.91	0.038	1.16
35	2.43	0.037	1.81
36	2.16	0.02	0.7
37	4.07	0.034	0.75
38	2.98	0.062	1.69
39	2.43	0.041	2.41
40	1.32	0.034	1.16
41	3.47	0.057	2.64
42	2.04	0.024	0.78
43	3.37	0.036	0.74
44	4.54	0.029	1.14
45	2.89	0.037	7.13
46	4.34	0.04	0.62
47	4.46	0.046	1.06
48	5.23	0.045	0.86
49	2.33	0.033	1.29
50	2.86	0.021	2.12
51	3.09	0.027	1.16
52	2.96	0.053	3.65
53	3.74	0.034	1.09
54	3.59	0.056	3.32
55	3.31	0.048	3.04
56	2.22	0.026	2.29
57	2.01	0.032	0.55
58	1.6	0.035	1.07
59	4.92	0.054	0.36
60	5.04	0.065	2.21

*: As standard deviation was the dependent response variable in this CCD, values are reported as SD only.

**Table 7:** Coded variables for optimized formulation.

Variable	Optimized Value
PLGA (mg)	82.96
T80 (mL)	6.78
Pressure (psi)	11,000.00
Pass Number	1

**Table 8:** Observed and model-predicted values (and residuals) of SD Z-average, SD PDI, and SD zeta potential for the optimized X drug delivery system obtained by desirability-based optimization.

Response	Observed	Predicted	Residual
SD Z-average (nm)	1.5301	1.53	0.0001
SD PDI	0.01225	0.0096	0.00265
SD zeta potential (mV)	0.512	0.48	0.032

## Data Availability

The original contributions presented in this study are included in the Supplementary Material. Further inquiries can be directed to the corresponding author.

## Supplementary Materials

The following supporting information can be downloaded at: https://www.mdpi.com/article/10.3390/pharmaceutics18010025/s1, Supplementary Data S1: The particle size distribution of PMFZ is determined as intensity, number, volume, PDI, Z-Average, and zeta potential according to the CCD experimental matrix. Supplementary Data S2: Goodness-of-fit statistics (R^2^, adjusted R^2^, MS residual, and S) for the polynomial response surface models of SD of Z-average, SD of PDI, and SD of zeta potential at each pass number. Supplementary Data S3: Three-dimensional surface plots and contour plots for standard deviations of Z-average, PDI, and Zeta Potentials as a function of PLGA amount, T80, pressure, and pass.

## Abbreviations

The following abbreviations are used in this manuscript:

QbD	Quality by Design
PLGA	Poly (lactic-co-glycolic acid)
MFZ	Microfluidizer
PMFZ	PLGANM produced by the MFZ method
POW	PLGANM produced using the traditional oil-in-water method
T80	Tween 80
CCD	Central Composite Design
OPMFZ	Optimized PMFZ
o/w	oil-in-water
NM	Nano-Micelles
(CQAs)	Critical quality attributes
DPPH	2,2-Diphenyl-1-picrylhydrazyl
PDI	Poly Dispersity Index
