# Supplementary material for: Optimization of Microfluidizer-Produced PLGA Nano-Micelles for Enhanced Stability and Antioxidant Efficacy: A Quality by Design Approach
Source: Pharmaceutics. 2025 Dec 25;18(1):25. doi: 10.3390/pharmaceutics18010025 (PMC12844988; doi:10.3390/pharmaceutics18010025)
Supplement: Supplementary file 1 [file pharmaceutics-18-00025-s001.zip › pharmaceutics-3976925-supplementary.pdf]

pharmaceutics

Supplementary Materials: Optimization of Microfluidizer-Produced PLGA Nano-Micelles for Enhanced Stability and Antioxidant Efficacy: A Quality by Design Approach

Esma Nur Develi Arslanhan, Fatemeh Bahadori, Zahra Eskandari, Muhammed Zahid Kasapoglu and Erkan Mankan

SUPPLEMENTARY DATA S1

The particle size distribution of PMFZ is determined as intensity, number, volume, PDI, Z average, and zeta potential according to the CCD experimental matrix

Run order	Int(1)	Int(7)	Int(15)	Int(30)	Int(60)
1	116,2	117,7	121,4	116,8	117,3
2	135,1	128,5	126,9	128,3	127,4
3	130,7	131,6	130,7	131,3	128,8
4	132,8	129,8	131,4	128	128,6
5	138	130,6	126,9	145,8	134,7
6	123,6	121	124,6	120,6	133,1
7	143,1	139,3	133,7	139,9	135,1
8	146,5	146,1	144	145,6	139,7
9	147,8	141	142,8	143,8	140,1
10	133,7	128,8	127,9	133,5	131,9
11	138,8	138,4	141,7	135,9	141,7
12	135,6	149,1	138,9	137,3	138,2
13	152,6	151,8	138,9	141,4	142,2
14	147,3	142,5	141,7	138,7	146
15	151	140,3	144,4	140,9	141,4
16	133,6	135,5	133,1	150,4	138,7
17	125,5	127,9	122,5	132,5	128,6
18	136,1	135,6	133	133	132,5
19	136,6	133,5	133,7	133,6	136,9
20	139,6	140,3	140,9	134,5	132,1
21	196	195,3	198,5	190,3	169,6
22	134,5	131,8	130,4	125,5	135,7
23	133,2	132,2	127,6	127,3	133,2
24	132,6	135,1	129,4	132,7	124,9
25	136,1	138,4	132	151,2	144,9
26	130,9	140,5	134,8	155,2	148
27	133,8	139,4	128,6	140,1	140,1
28	143,1	147,8	139,2	143,1	142,3
29	139	136,6	136	130,8	125,8
30	130,9	131,2	142,7	144,6	137,1
31	131,8	124,4	134,4	129,7	129,2
32	128,7	134,8	122,7	137,3	131
33	134,3	134,6	133,3	133,9	131,4
34	133,8	140	129,9	131	133,1
35	140,6	139	135,2	140	146,5
36	145,8	141,5	137,2	141,6	142,5
37	141,5	142,7	147,7	148,8	134,4
38	136,6	133,8	139,2	134,6	137,2
39	140,2	134,4	130	136,6	134,9
40	131,8	131	128,5	139,5	130,5
41	123,3	122,9	119,9	124,8	124
42	139,6	127,9	136	133,9	130,3
43	144,7	147,4	140,9	137,4	134,4
44	125	135,2	123,6	133,5	129,4
45	140,8	137,2	146	141,3	134,2
46	143,4	138,3	135,6	148,1	132
47	138,7	133,4	136,1	141,8	138
48	157,1	143,5	135,1	157,5	148,7
49	139,6	137,3	137	137,3	143
50	145,1	139,9	144,5	145,8	140,9
51	136,7	138,1	141,5	134,1	134,1
52	124	129,9	126,2	134,1	117,1
53	141,6	143,3	143,4	136	134,2
54	145,6	143,1	139,4	140,1	135,6
55	150,2	145,1	149,6	146,2	157,3
56	139,6	136,8	136,1	134,1	132
57	132,8	131,9	136,6	136,6	139,4
58	136,9	142,9	139,2	136,9	137,3
59	143,8	148,4	140,5	151,6	133,5
60	139,7	144,2	139,4	146,5	148,5

Run order	Num(1)	Num(7)	Num(15)	Num(30)	Num(60)
1	73,69	73,86	82,41	88,76	109,4
2	88,16	86,51	91,11	99,35	91,24
3	94,88	82,72	94,11	97,85	99,97
4	97,43	94,33	100,8	92	106,1
5	81,04	92,79	102,9	89,99	85,44
6	82,47	87,79	85,47	92,8	73,65
7	107,8	85,65	108	95,8	103,6
8	110,4	90,29	106	99,87	101,4
9	87,91	91,66	90	95,9	99,52
10	92,38	89,76	98,79	84,13	88,45
11	96,37	91,18	84,62	108	90,7
12	111,80	85,5	103,5	104,7	105,3
13	85,74	95,22	113,9	106,7	106,2
14	98,4	96,82	108,9	97,68	94,61
15	103,8	111	112,3	115,2	111,8
16	97,2	105,8	104	102,5	85,58
17	96,99	90,41	89,52	83,77	93,47
18	97,76	77,66	92,74	91,38	97,9
19	104	102,2	103,8	95,25	85,26
20	103,2	85,96	103,2	97,89	105,1
21	155,3	152,7	144,1	127,7	130,6
22	96,56	96,16	91,02	102,1	95,27
23	100,5	87,34	100,7	99,78	96,4
24	91,74	88,02	90,08	84,72	92,11
25	107,5	88,85	98,3	96,54	87,42
26	108,2	88,5	110,5	94,17	90,95
27	100,3	91,49	91,07	89,95	86,08
28	109,1	109,5	111,4	113,3	108,5
29	83,05	86,88	89,6	90,35	90,2
30	102,2	97,34	96,69	96,94	85,48
31	91,05	89,64	85,27	102,8	95,4
32	98,66	86,75	97,92	87,41	91,8
33	100,1	91,76	101,1	108	89,01
34	99,95	82,23	100,4	92,46	92,5
35	97,73	106,5	104,9	107,8	87,88
36	112,1	107,3	112,2	108	100,6
37	118,9	110,4	94,22	89,6	104,3
38	107,4	93,12	104,7	106,9	74,78
39	90,17	103,6	101,9	103	96,76
40	102,3	98,12	89,73	76,6	90,84
41	100,5	84,86	85,56	79,16	80,91
42	82,25	96,4	88,13	101,5	98,27
43	98,93	105,8	105,5	103,5	104,3
44	98,6	84,04	83,25	82,34	76,17
45	102,8	100,9	85,81	82,16	94,86
46	104,9	105,9	87,5	99,6	96,79
47	100,9	104,4	94,38	114,3	95,27
48	95,08	91,53	114,5	103,8	83,65
49	115,6	112,1	97,99	110,9	96,19
50	107,8	103	104,7	103,1	96,32
51	104,2	91,57	100,3	100,8	92,42
52	91,18	86	94,5	85,96	97,68
53	109,3	94,15	97,22	107,8	93,21
54	112	99,59	107,6	106,1	94,55
55	109,7	108,9	106,7	117,3	120,5
56	104,2	92,63	95,14	100,5	98,29
57	109,6	104	96,85	99,9	107,7
58	105,1	90,34	98,3	103,8	97,59
59	104,2	91,24	116,8	106,6	95,26
60	110,3	91,95	106,3	118,5	95,86

Run order	Vol(1)	Vol(7)	Vol(15)	Vol(30)	Vol(60)
1	96,07	96,88	104,6	104,8	99,74
2	117	111,4	112,7	117,7	113,2
3	117,6	111,6	117,1	119,2	118,3
4	120,2	116,6	120,5	114	120,9
5	115,3	116,4	118,3	126,9	115
6	105,8	106,9	108,3	109,5	107,2
7	132,8	119,2	125,5	124,9	124,6
8	136,6	126,9	132,7	131,6	127,3
9	126,8	123,4	124,2	127,9	127
10	118,2	113,4	117,2	113,6	114,9
11	124,2	121,4	119,8	127,2	123,7
12	128,4	126,4	127,6	126,8	127,8
13	129	133,8	131,7	130,9	131,4
14	131,9	127,5	132,1	124,9	128,7
15	137,7	131,8	135,7	133,8	133,1
16	120,7	126	123,3	136,6	118,8
17	114,7	112,9	108,8	112,7	115,3
18	122,8	111,6	117,9	117,1	120,3
19	125,9	122,9	123,7	119,7	117,1
20	128	119,7	129	121,8	123,1
21	194,2	193,1	195	183	163,7
22	121	119	115,1	117	121,3
23	121,9	115	117,8	117,2	120,2
24	117,1	117,1	113,7	113,6	112
25	127,2	120,2	120	134,1	123,15
26	123,5	121,3	127,3	137	128,8
27	122,2	122,2	113,8	121,6	120,1
28	133,4	137,4	131,1	135	132,5
29	117,3	118	118,4	115	111,4
30	120,8	119,1	127,6	129,2	111,1
31	116	110,1	115,1	120,2	116,7
32	117,7	116,1	113,1	118,3	116,1
33	122,6	118,8	122,2	125,7	114,8
34	122,1	116,9	119,4	116,3	117,9
35	124,1	129	125,3	130,4	126,2
36	136,8	131,3	129,8	131,7	129,3
37	135,6	133,5	129,8	129,1	124,4
38	127,5	118,8	128,3	125,7	110,2
39	122,2	124,2	120	125,5	121,6
40	121,5	119,3	112,9	113,1	115,3
41	114,7	107,1	105,1	104,1	105,3
42	117	116,2	118	116,3	118,8
43	130,2	135,2	130	126,3	125,3
44	115	115,2	106,2	112,6	106,1
45	128,7	125,3	124,6	117,8	120,1
46	131,6	128,2	116,9	133,4	119,3
47	126,2	123,7	121,1	134,3	123,2
48	138,5	125,2	128,9	143,2	125,5
49	132,9	129,8	123,8	129,4	127,4
50	134,4	128,2	132,3	132,9	126
51	126,2	120,9	128,3	122,7	118,6
52	110,8	112,3	114,2	115,6	109,2
53	132,3	126,5	128,4	127,2	119,2
54	136,5	129,4	129,8	129,7	120,9
55	139,5	134,8	137,5	138,9	149,4
56	128,3	120,7	121,7	122,6	120
57	125,4	122,5	122,9	124,4	129,8
58	126,7	123,9	125,6	126,1	123,7
59	131,7	128,8	134	139,4	119,7
60	131,1	126,3	129,2	139,5	131,5

Run order	Z-Ave(1)	Z-Ave(7)	Z-Ave(15)	Z-Ave(30)	Z-Ave(60)
1	105,6	105,9	111,8	111	109,4
2	121,9	117	118	122,3	117,8
3	122	118,2	121,8	121,7	119,9
4	121,3	121,2	122,7	119,2	123,1
5	122,4	120,7	119,4	123,4	124,1
6	112,5	112,6	114,3	113,8	115,8
7	133,6	124,4	127,9	127,7	128
8	134,4	130,4	132,4	133,3	129,1
9	130,8	127,6	127,5	136,5	130,7
10	121,3	118,4	121,5	120	119,9
11	126,6	125,01	124,9	129,8	130,3
12	131,3	130,5	129,4	129,8	130
13	132	135,2	134,1	131,2	133,3
14	135,2	130,5	132,1	131,2	132,5
15	137,5	127,5	136,6	135,2	135,4
16	125,1	127,9	126,1	137,7	124,3
17	117,3	117,3	115,7	118,9	119,1
18	124,4	119,8	121,8	121,9	124,4
19	126,6	126,7	126,7	122,4	123
20	128,9	124,9	130,1	125,2	126,5
21	180,7	179,5	176,4	168,7	162,3
22	123,4	123,9	119,7	120,5	124,3
23	124,4	121,5	121,5	120,4	124,3
24	121,1	120,7	118,2	119,2	117,5
25	129,1	125,2	124,3	137,8	126,9
26	125,9	126	130,9	134,5	132,3
27	124,4	126	124	126	124,7
28	134,9	137,5	132,9	136,3	135
29	123,7	123,1	122,5	119,5	119,7
30	123,3	123,9	130	131,3	122,3
31	122	118,6	121,3	122,7	120,3
32	121,3	122,3	115,9	123,3	121,1
33	125,8	124,3	124,9	129,4	120
34	124,8	123,1	122,9	120,6	120,2
35	126,8	130,6	127,8	132,9	130,5
36	136,2	132,3	130,8	133,3	131,2
37	138	134,4	132	132,5	126,8
38	127,8	122	127,4	128,5	123,1
39	128,9	127,7	123,9	127,4	123,5
40	122,8	122,1	120,8	120,7	119,4
41	119,9	116,4	111,7	112,2	113
42	124	119,6	121,6	124,7	121,9
43	134,1	134,8	131,8	127,8	127,7
44	117,9	125,2	114,8	119	113,6
45	129,3	129	126,4	123,7	123,1
46	134,3	129,5	127,4	133,6	123,9
47	127,8	126,9	125	135,8	125,1
48	138	130,2	130	141,3	130,9
49	132,7	130,8	127,1	132,3	129,1
50	134,8	130,5	132,9	133	127,4
51	128,1	125,1	130,4	125,4	122,3
52	117,8	118,3	118,8	121,5	113,3
53	133,8	129,4	129,1	129,4	123,3
54	136,1	130,8	132	132,7	126,2
55	140,2	134,2	137	138,6	143
56	129	124,5	126,1	125,7	123
57	128,5	125,9	125,6	128	130,5
58	127,6	129,3	128,8	128,2	125,2
59	132,2	131,4	135,1	138,8	125,5
60	133,3	129,5	129,7	139,4	139,8

Run order	PdI(1)	PdI(7)	PdI(15)	PdI(30)	PdI(60)
1	0,112	0,095	0,07	0,014	0,103
2	0,093	0,105	0,123	0,011	0,076
3	0,054	0,098	0,054	0,089	0,094
4	0,128	0,051	0,08	0,129	0,036
5	0,103	0,065	0,08	0,056	0,144
6	0,128	0,063	0,081	0,056	0,116
7	0,056	0,099	0,022	0,081	0,019
8	0,119	0,099	0,059	0,072	0,086
9	0,1	0,083	0,122	0,202	0,036
10	0,108	0,072	0,023	0,092	0,088
11	0,117	0,091	0,115	0,011	0,153
12	0,024	0,074	0,06	0,028	0,045
13	0,123	0,102	0,018	0,087	0,042
14	0,128	0,074	0,08	0,144	0,133
15	0,085	0,177	0,036	0,009	0,003
16	0,117	0,05	0,04	0,128	0,088
17	0,073	0,1	0,128	0,099	0,078
18	0,122	0,103	0,101	0,074	0,042
19	0,095	0,02	0,031	0,102	0,092
20	0,091	0,12	0,085	0,054	0,002
21	0,079	0,066	0,113	0,121	0,223
22	0,095	0,031	0,08	0,002	0,105
23	0,067	0,055	0,019	0,046	0,052
24	0,081	0,113	0,092	0,099	0,028
25	0,041	0,085	0,144	0,165	0,115
26	0,017	0,094	0,031	0,113	0,089
27	0,08	0,09	0,164	0,124	0,097
28	0,039	0,064	0,021	0,021	0,011
29	0,095	0,08	0,1	0,09	0,139
30	0,058	0,025	0,078	0,086	0,109
31	0,133	0,148	0,083	0,041	0,067
32	0,048	0,137	0,065	0,096	0,092
33	0,042	0,048	0,051	0,021	0,084
34	0,07	0,111	0,027	0,116	0,111
35	0,102	0,05	0,041	0,017	0,097
36	0,073	0,062	0,039	0,032	0,076
37	0,041	0,031	0,101	0,098	0,042
38	0,086	0,099	0,121	0,013	0,185
39	0,119	0,014	0,076	0,068	0,106
40	0,09	0,067	0,136	0,125	0,058
41	0,041	0,119	0,121	0,186	0,063
42	0,094	0,063	0,113	0,074	0,052
43	0,106	0,118	0,048	0,076	0,035
44	0,063	0,139	0,12	0,102	0,118
45	0,103	0,03	0,118	0,117	0,079
46	0,115	0,066	0,172	0,101	0,138
47	0,09	0,02	0,11	0,007	0,083
48	0,121	0,139	0,021	0,102	0,1
49	0,05	0,04	0,063	0,014	0,104
50	0,07	0,053	0,101	0,098	0,098
51	0,057	0,122	0,071	0,061	0,095
52	0,141	0,085	0,031	0,08	0,003
53	0,031	0,088	0,105	0,029	0,078
54	0,071	0,087	0,157	0,013	0,132
55	0,041	0,092	0,123	0,042	0,147
56	0,09	0,112	0,051	0,054	0,07
57	0,01	0,01	0,08	0,04	0,068
58	0,082	0,133	0,062	0,054	0,118
59	0,088	0,104	0,01	0,069	0,158
60	0,015	0,1	0,083	0,03	0,18

Run order	Zeta(15)	Zeta(21)	Zeta(30)	Zeta(60)
1	-7	-9,01	-7,77	-17,5
2	-5,27	-5,06	-3,14	-3,97
3	-0,71	-3,17	-2,57	-5,25
4	-3,99	-3,76	-3,01	-3,97
5	-4,38	-3,53	-2,68	-3,59
6	-4,23	-2,69	-3,21	-3,13
7	-5,7	-4,12	-3,67	-7,8
8	-4,02	-3,92	-4,46	-6,56
9	-4,34	-4,19	-4,69	-5,04
10	-5,24	-5,33	-6,52	-3,16
11	-3,44	-2,35	-3,88	-5,78
12	-2,8	-2,42	-2,91	-4,13
13	-2,04	-1,92	-2,28	-4,66
14	-4,75	-4,62	-4,19	-2,59
15	-4,1	-4,08	-3,83	-6,52
16	-4,29	-4,61	-3,56	-5,63
17	-4,54	-2,97	-2,82	-2,25
18	-6,21	-6,02	-3,8	-7,43
19	-5,08	-3,74	-3,46	-5,33
20	-6,16	-4,43	-4,06	-7,88
21	-3,37	-2,75	-2,98	-1,86
22	-4,23	-4,27	-4,02	-8,08
23	-4,87	-3,76	-3,92	-5,03
24	-5,49	-3,16	-2,5	0,136
25	-7,64	-4,93	-4,65	-3,42
26	-1,91	-2,49	-1,37	-2,32
27	-3,61	-4,29	-2,96	-4,53
28	-4,66	-4,86	-4,48	-5,94
29	-3,29	-5,47	-4,55	-4,58
30	-4,25	-7,1	-4,05	-5,07
31	-5,1	-6,46	-7,95	-3,67
32	-8,34	-8,04	-9,56	-14,1
33	-8,62	-6,3	-4,74	-3,79
34	-6,1	-4,73	-5,94	-3,61
35	-5,34	-3,26	-6,49	-7,46
36	-4,68	-5,05	-4,71	-3,46
37	-5,44	-4,12	-4,01	-5,28
38	-4,81	-5,77	-3,61	-7,6
39	-4,21	-4,84	-6,2	-9,6
40	-4,67	-6,37	-5,7	-3,72
41	-0,708	-5,06	-3,31	-6,92
42	-5,13	-5,55	-3,74	-5,06
43	-3,79	-2,32	-3,16	-2,22
44	-4,7	-3,77	-2,81	-5,44
45	-3,05	-8,72	-10,8	-20,2
46	-5,35	-6,13	-6,69	-5,47
47	-7,93	-5,97	-5,51	-6,21
48	-5,41	-5,26	-4,27	-6,36
49	-6,8	-4,56	-4,84	-7,05
50	-6,43	-9,44	-4,88	-5,03
51	-8,52	-6,12	-6	-6,79
52	-6,78	-0,208	-7,08	-8,29
53	-7,27	-5,2	-6,47	-4,94
54	-11,6	-6,31	-7,54	-3,61
55	-4,87	0,213	-4,37	-7,01
56	-0,741	-6,32	-4,02	-3,88
57	-5,31	-5,21	-4,47	-5,81
58	-5,15	-7,43	-6,29	-7,33
59	-5,03	-4,78	-4,71	-4,17
60	-3,77	-4,55	-4,94	-8,73

SUPPLEMENTARY DATA S2

Goodness-of-fit statistics (R^2, adjusted R^2, MS residual and S) for the polynomial response surface models of SD of Z-average, SD of PDI and SD of zeta potential at each pass number.

SD of Z-average (SDV- Z-AVE)

**Pass**	**n**	**R^2**	**Adjusted R^2**	**MS residual**	**S**
1	20	0.513	0.076	2.101820	1.4498
3	20	0.302	−0.327	3.080969	1.7553
5	20	0.647	0.330	0.674441	0.8212

SD of PDI (SDV-PDI)

**Pass**	**n**	**R^2**	**Adjusted R^2**	**MS residual**	**S**
1	20	0.380	−0.177	0.000173	0.0132
3	20	0.781	0.583	0.000079	0.0089
5	20	0.855	0.724	0.000032	0.0057

SD of Zeta Potential (SDV-ZETA)

**Pass**	**n**	**R^2**	**Adjusted R^2**	**MS residual**	**S**
1	20	0.269	−0.390	3.532598	1.8795
3	20	0.451	−0.044	0.575321	0.7585
5	20	0.543	0.131	0.690618	0.8310

The observed negative adjusted R^2 values for some responses arise from modeling inherently low-variance outcomes (standard deviations), where limited signal-to-noise ratio leads to reduced explained variability.

SUPPLEMENTARY DATA S3

The response surface models used to optimize nanoparticle formulations.

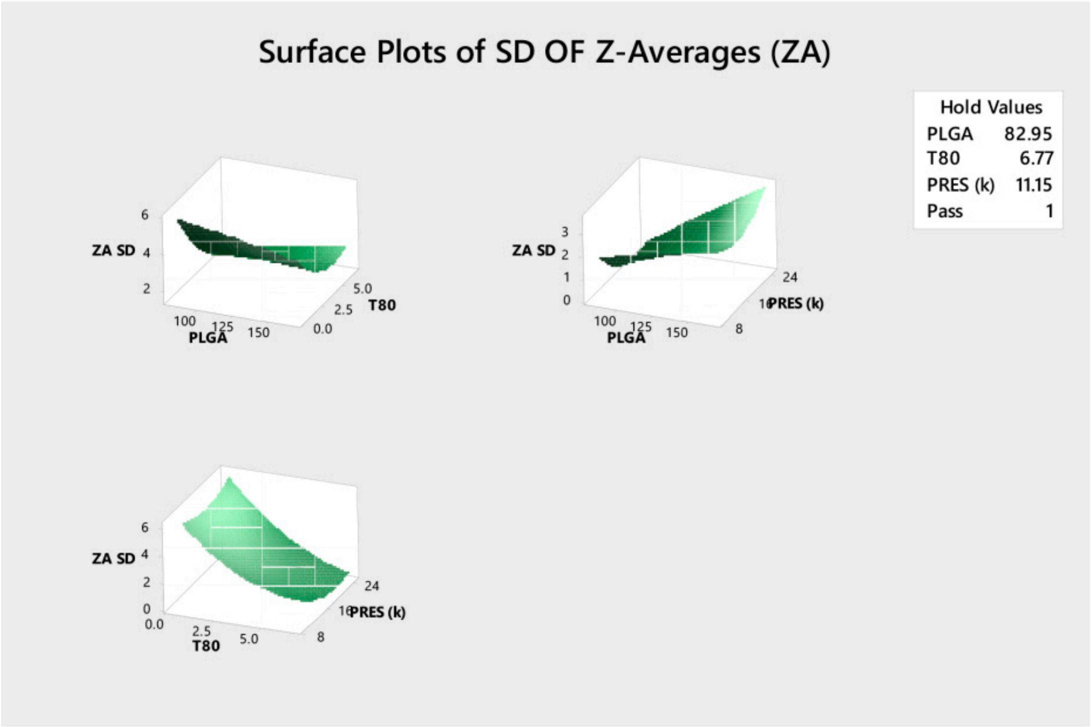

Figure S1. Response surface plot illustrating the dependence of particle size (Z-average) on formulation parameters. The figure highlights that an optimal balance of Tween 80 and PLGA leads to a lower size variation, with excessive concentrations causing instability.

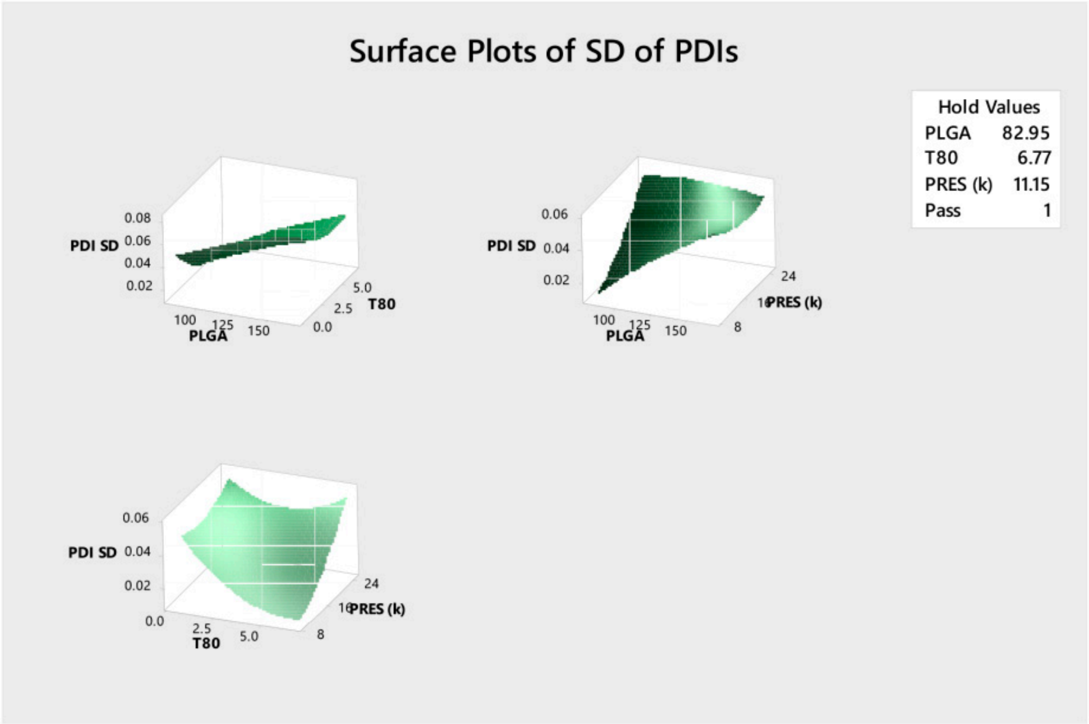

Figure S2. Response surface plot showing the effect of formulation parameters on the polydispersity index (PDI). The model suggests that a specific range of PLGA and Tween 80 concentrations results in a more monodisperse population.

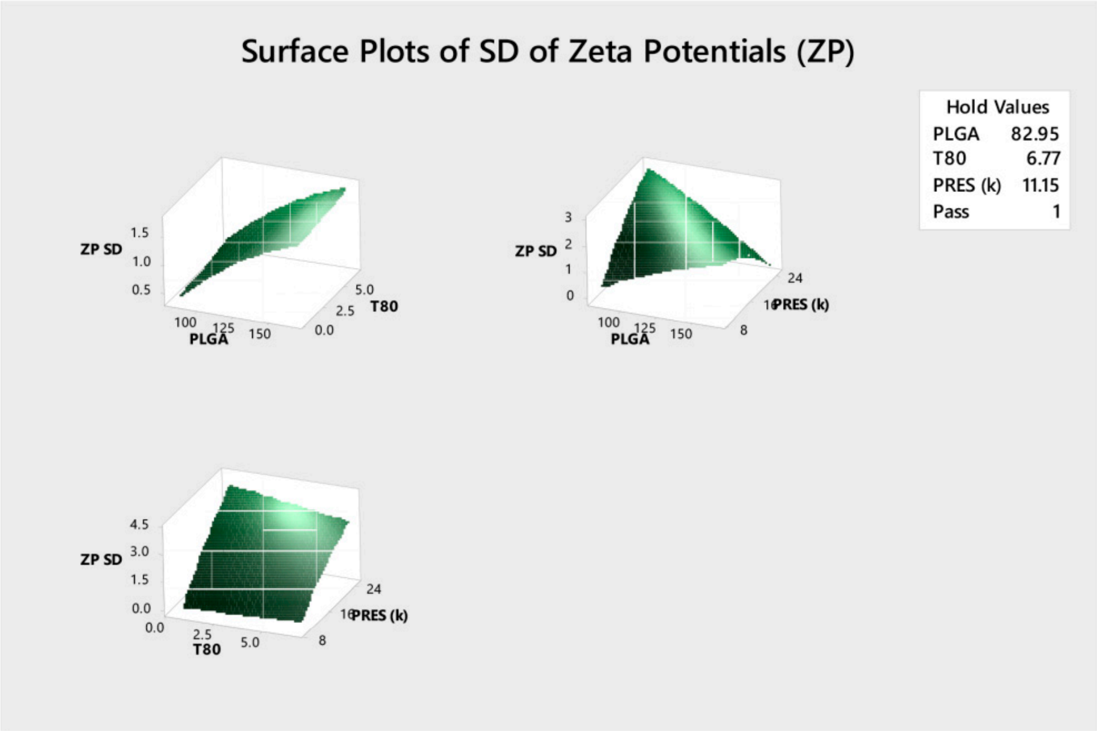

Figure S3. Response surface plot displaying the relationship between formulation parameters and zeta potential variability. Stability is maximized when Tween 80 and PLGA concentrations remain within a controlled range, reducing surface charge fluctuations.
